# Acute systemic inflammatory response to lipopolysaccharide stimulation in pigs divergently selected for residual feed intake

**DOI:** 10.1186/s12864-019-6127-x

**Published:** 2019-10-11

**Authors:** Haibo Liu, Kristina M. Feye, Yet T. Nguyen, Anoosh Rakhshandeh, Crystal L. Loving, Jack C. M. Dekkers, Nicholas K. Gabler, Christopher K. Tuggle

**Affiliations:** 1 Department of Animal Science, Iowa State University, 2258 Kildee Hall, Ames, IA 50011 USA; 2 Interdepartmental Immunobiology, Department of Animal Science, Iowa State University, 2258 Kildee Hall, Ames, IA 50011 USA; 3 Department of Mathematics and Statistics, Old Dominion University, Norfolk, VA 23529 USA; 4 Department of Animal and Food Sciences, Texas Tech University, Lubbock, TX 79409 USA; 5 Food Safety and Enteric Pathogens Research Unit, National Animal Disease Center, ARS, USDA, 1920 Dayton Ave, Ames, IA 50010 USA; 6 Department of Animal Science, Iowa State University, 239 Kildee Hall, Ames, IA 50011 USA; 7 Department of Animal Science, Iowa State University, 2255 Kildee Hall, Ames, IA 50011 USA

**Keywords:** *Sus scrofa*, Lipopolysaccharide, Systemic inflammation, Residual feed intake, RNA-seq

## Abstract

**Background:**

It is unclear whether improving feed efficiency by selection for low residual feed intake (RFI) compromises pigs’ immunocompetence. Here, we aimed at investigating whether pig lines divergently selected for RFI had different inflammatory responses to lipopolysaccharide (LPS) exposure, regarding to clinical presentations and transcriptomic changes in peripheral blood cells.

**Results:**

LPS injection induced acute systemic inflammation in both the low-RFI and high-RFI line (*n* = 8 per line). At 4 h post injection (hpi), the low-RFI line had a significantly lower (*p =* 0.0075) mean rectal temperature compared to the high-RFI line. However, no significant differences in complete blood count or levels of several plasma cytokines were detected between the two lines. Profiling blood transcriptomes at 0, 2, 6, and 24 hpi by RNA-sequencing revealed that LPS induced dramatic transcriptional changes, with 6296 genes differentially expressed at at least one time point post injection relative to baseline in at least one line (*n* = 4 per line) (|*log*_2_(fold change)| ≥ *log*_2_(1.2); *q* < 0.05). Furthermore, applying the same cutoffs, we detected 334 genes differentially expressed between the two lines at at least one time point, including 33 genes differentially expressed between the two lines at baseline. But no significant line-by-time interaction effects were detected. Genes involved in protein translation, defense response, immune response, and signaling were enriched in different co-expression clusters of genes responsive to LPS stimulation. The two lines were largely similar in their peripheral blood transcriptomic responses to LPS stimulation at the pathway level, although the low-RFI line had a slightly lower level of inflammatory response than the high-RFI line from 2 to 6 hpi and a slightly higher level of inflammatory response than the high-RFI line at 24 hpi.

**Conclusions:**

The pig lines divergently selected for RFI had a largely similar response to LPS stimulation. However, the low-RFI line had a relatively lower-level, but longer-lasting, inflammatory response compared to the high-RFI line. Our results suggest selection for feed efficient pigs does not significantly compromise a pig’s acute systemic inflammatory response to LPS, although slight differences in intensity and duration may occur.

## Background

Feed efficiency in pigs is a trait of economic, environmental and societal importance. One increasingly accepted measure of feed efficiency is residual feed intake (RFI), which is defined as the difference between an individual animal’s observed and expected feed intake for growth and maintenance [1]. Thus pigs with a low RFI are more feed efficient than those with a high RFI. Pilot studies of divergent selection for RFI in pigs showed that RFI responds well to genetic selection [2–4].

Compared to high-RFI pigs, pigs selected for low RFI have reduced feed intake, but similar rate of growth [2–4]. This difference occurs likely because the low-RFI pigs are more efficient in allocating resources for production and maintenance [5]. The immune response is a nutrient- and energy-demanding biological process and directly relates to pig health and performance [6, 7]. Thus, one interesting question is whether improving feed efficiency by selection for low RFI affects the animal’s ability to respond to immune challenges. Based on resource allocation theory [5], selection for high feed efficiency is expected to compromise the animal’s capacity to handle immune stimulation, such as the response that occurs during infectious diseases [8]. This has been confirmed in studies on chickens and beef cattle, where selection for increased feed efficiency indeed negatively affected their immune system [9].

Several experiments have investigated the potential side effects of selection for divergent RFI phenotypes on the immune response in pigs. First, a study of healthy pigs from the divergently selected RFI lines at Iowa State University (ISU) [2–4], from which representatives were used in the current study, showed that the low-RFI line had lower numbers of monocytes, lymphocytes, and basophils, but a higher hemoglobin concentration and red blood cell volume compared to the high-RFI line [10]. Second, based on results from an experimental infection with the porcine reproductive and respiratory syndrome virus (PRRSV) in pigs from the ISU RFI lines [2–4], Dunkelberger et al. [11] reported that pigs from the low-RFI line had a lower viral RNA load in the blood, a faster humoral immune response to PRRSV, and were less affected in terms of reduced growth rate than pigs from the high-RFI line. Third, in a parallel divergent selection experiment conducted at the French National Institute for Agricultural Research (INRA), the low-RFI line had a lower basal expression of many genes involved in immune and inflammatory response than the high-RFI line [12, 13]. Fourth, to test the immune response in the divergently selected RFI lines developed by INRA, piglets from both lines were challenged with the Complete Freund’s Adjuvant (CFA) to induce a non-infectious pneumonia [14–17]. This work showed that both RFI lines handled the inflammatory challenge similarly, but did so by adopting different metabolic strategies [15]. Interestingly, the protein abundance of inflammatory cytokines was lower in the low-RFI line in multiple tissues involved in the immune response 1 week after CFA injection [16]. Lastly, Vigros et al. [18] examined the expression profiles of a set of target genes related to intestinal inflammation in pigs with extremely divergent RFI phenotypes, both before and after an ex vivo LPS exposure of ileal and colonic tissue explants. No differentially expressed genes were detected in the un-stimulated explants. However, the mRNA levels of several proinflammatory cytokines (IL8, IL1, IL6, TNFα, IFNγ) and SOCS3 were lower in the low-RFI than the high-RFI explants following LPS challenge [18]. These authors proposed that low-RFI pigs may adopt an energy saving mechanism during intestinal responses to an immune challenge [18]. Taken together, although no significant negative side effects of selection for increased feed efficiency based on reduced RFI on the immune response in pigs have been detected, little is known about the effect of selection for RFI on the global transcriptomic profiles during the course of acute systemic inflammatory response in pigs.

As a major component of the outer membrane of most gram-negative bacteria [19], LPS has been widely used in vertebrates as an inflammatory immunostimulant. In vertebrates, LPS induces inflammatory response mainly via the TLR4-dependent NFκB signaling pathway [20, 21], although a TLR4-independent host response to LPS has also been identified [22]. Thousands of genes, including many pro-inflammatory and anti-inflammatory cytokines and chemokines, have been shown to be involved in the LPS-induced inflammatory response in multiple vertebrates, including pigs [23–28]. LPS stimulation can cause many physiological and behavioral changes, including elevated body temperature, dramatic hemodynamic, increased cytokine levels, reduced feed intake, and altered metabolism [29–32].

The objective of this study was to determine whether divergent selection for RFI significantly affects the pig’s systemic inflammatory/immune response to LPS. We induced acute systemic inflammatory response by intramuscular injection of LPS in two lines that were divergently selected for RFI [2, 33], and then measured changes in body temperature, complete blood count (CBC), plasma cytokine levels, and peripheral blood transcriptome over time in these lines by using RNA-seq. We detected a few minor differences between the two RFI lines’ systemic inflammatory responses triggered by LPS, although acute responses were substantial in both lines. Our work suggests that the peripheral blood gene expression of low-RFI pigs is only slightly different from that of high-RFI pigs in response to an acute inflammatory challenge.

## Methods

### Animals, experimental design, and sample collection

**Fig. 1:**
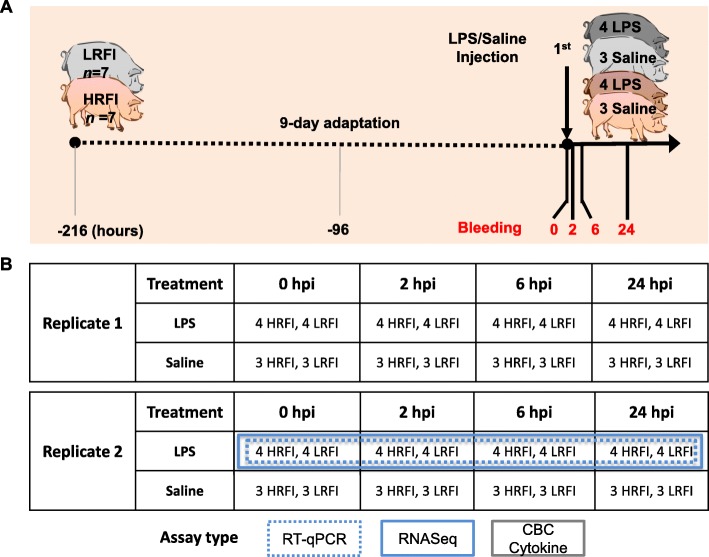
Experimental design and blood sample collection. **a** The experiment was performed in two replicates. Shown here is one of the two replicates. Fourteen gilts with a similar initial body weight (BW) from the low-RFI and high-RFI lines (*n* = 7 per line) were randomly selected and used for each replicate. Pigs were housed in individual metabolism crates, and had ad libitum access to water, but were restricted to feed intake. After a 9-day adaptation period, pigs within a line were randomly assigned to either a control (*n* = 6, three pigs per line) or LPS treatment (*n* = 8, four pigs per line) group. Pigs in the treatment group were then challenged with LPS via intramuscular injection of 30 μg/kg BW of LPS from *E. coli* O55:B5 dissolved in an endotoxin-free, sterile saline solution at baseline (0 hpi). Pigs in the control group were injected with an equivalent volume of endotoxin-free, sterile saline solution at the equivalent time. The rectal temperature of each pig was measured at 0, 2, 4, 6, and 24 hpi. At 0, 2, 6, and 24 hpi, blood samples were collected from the jugular vein into Tempus™ Blood RNA tubes for long-term storage at − 80 °C, into EDTA tubes for CBC tests and cytokine assays. For more details, see Materials and Methods. **b** Shown are the numbers of animals with blood samples collected from the two replicates at 0, 2, 6, and 24 hpi and the types of assays performed on different samples. Only blood samples collected from the LPS treated group from Replicate 2 were used for RT-qPCR and RNA-seq. Blood samples collected from all animals from both replicates were used for CBC tests and cytokine assays. The images of pigs were created by one of the co-authors, Anoosh Rakhshandeh and agreed to be published here. HRFI, high-RFI line; LRFI, low-RFI line

The pigs for this study were exclusively from Generation 8 of the Yorkshire pig lines divergently selected for RFI at ISU [2, 33], which are developed and owned by Dr. Jack CM Dekkers, one of the co-authors. The experiment was performed in two independent replicates of a 2-by-2 factorial design with repeated measures. The sample size, LPS dosage, and injection route of LPS were determined by referring to our previous study [34]. Figure 1a shows the experimental design for one replicate. A total of 28 gilts with an initial body weight (BW) of 63 ± 4 kg from the low-RFI and high-RFI lines (*n* = 14 per line) were randomly selected and utilized for the two replicates. Pigs were housed in randomly assigned individual metabolism crates, had ad libitum access to water, and were fed a corn-soy-based diet twice daily (8,00 am and 5:00 pm), with feed restriction (1.5 kg/day), as previously described [35]. After a 9-day adaptation period, pigs within each line were randomly assigned to either a control (*n* = 6, three pigs per line) or LPS treatment (*n* = 8, four pigs per line) group. Pigs in the treatment group were then challenged with LPS using an established method [34] via intramuscular injection of 30 μg/kg BW of LPS from *E. coli* O55:B5 (Sigma-Aldrich, St. Louis, MO, USA) dissolved in a endotoxin-free, sterile saline solution at baseline, 0 h post injection (hpi). Pigs in the control group were injected with an equivalent volume of endotoxin-free, sterile saline solution at the equivalent time. The rectal temperature of each pig was measured at 0, 2, 4, 6, and 24 hpi. At 0, 2, 6, and 24 hpi, blood samples were collected from the jugular vein into Tempus™ Blood RNA tubes (Life Technologies, Grand Island, NY, USA) for long-term storage at − 80 °C, into EDTA tubes (BD, Franklin Lakes, NJ, USA) for complete blood count tests and cytokine assays. Injection, temperature measurement, and blood collection followed the same order which was the predefined order of metabolism crates in the pen rooms. The sampling time points were determined by referring to a previous study where time series response of humans to LPS were investigated [24]. Figure 1b show the number of blood samples collected at each time points from each replicate. At 168 hpi, all pigs were anesthetized by intraperitoneal injection of sodium pentobarbital (100 mg/kg BW), and exsanguinated by cutting their carotid and jugular vessels.

### RNA preparation

Total RNA was extracted from the Tempus tube samples from Replicate 2 of the treatment groups for both pig lines by using preserved blood RNA purification kit I (Norgen Biotek Corp, Thorold, Ontario, CA) per the manufacturer’s instructions. On-column DNA digestion was performed using DNase I (Qiagen, Valencia, CA, USA). Globin transcripts (HBB and HBA) were depleted by following an RNase H-mediated method [36]. The quantity and integrity of the RNA were monitored by using Nanodrop 2000 (Thermo Scientific, Waltham, MA, USA) and Bioanalyzer 2100 (Agilent Technologies, Santa Clara, CA, USA) before and after globin depletion. The efficiency of globin depletion of each sample was checked by conventional RT-qPCR with ACTB and GAPDH as the internal controls. Globin depletion efficiencies for all RNA samples were above 85%. Metadata, including RNA integrity numbers (RINs) and concentration of RNA post globin depletion, CBC, and sequencing batches are available in Additional file 1: Table S1.

### Complete blood count (CBC) tests and plasma cytokine assays

CBC tests were performed for all except five clotted samples by the Pathology Lab, College of Veterinary Medicine at ISU as described [10]. Eight cytokines (IFNα, IFNγ, IL1β, IL4, IL6, IL8, IL10 and TNFα) in the all 112 plasma samples were assayed by using Aushon SearchLight Arrays for pig cytokines (Aushon BioSystems, Billerica, MA, USA) per the manufacturer’s instructions.

### Verification of transcriptional response to LPS stimulation by RT-qPCR

Primers for 47 candidate genes, which were porcine orthologs of human and murine genes responsive to LPS stimulation or important for Gram-negative sepsis control and resolution, and three house-keeping genes (ACTB, RPL32 and GAPDH), were designed and synthesized by Fluidigm Corporation (Fluidigm Corporation, San Francisco, CA, USA) such that two primers of each pair were separated by exon-exon boundaries and could amplify all known isoforms of the target gene, if possible. The specificities of the primer pairs were tested by conventional RT-qPCR using the DNA Engine Opticon 2 System (BioRad, Hercules, CA, USA) and only primer pairs that gave single peaks in melting curve analyses were kept. Additionally, the qPCR was performed without the melt curve analysis step and the amplicons were visualized on a 2% agarose gel for doublets, significant primer dimers, and confirmation of the amplicon size. Primer pairs producing amplicons of unexpected sizes were removed from the study. In total, this quality control scheme resulted in 36 and two primer pairs for genes of interest and internal controls (RPL32 and GAPDH) meeting the requirements, respectively (Additional file 2: Table S2). The 32 RNA samples from Replicate 2 of the treatment group were used for Fluidigm RT-qPCR without globin depletion. By following the Fluidigm user guide for Real-Time PCR analysis [37], real time-qPCR was done on a 48.48 dynamic array chip (Fisher Scientific, Pittsburgh, PA, USA), along with reactions for assessing primer amplification efficiency, using the Biomarker HD system (Fluidigm Corporation, San Francisco, CA, USA). Data were analyzed with the Fluidigm Real-Time PCR analysis software using the default settings, to obtain raw *C*_*t*_ (cycle of threshold) values. Since the expression levels of internal controls were not very stable for individual pigs during the time course, RT-qPCR data were analyzed by using the R package *MCMC.qpcr* (version 1.2.3) [38]. With this method, internal reference genes are not mandatory but can be incorporated as Bayesian priors or as trackers of global effects when template abundances correlate with experimental conditions [38]. Briefly, *C*_*t*_ values were converted into the copy number of templates by incorporating the amplification efficiencies of the primers and then analyzed using a generalized linear mixed model, which assumed the copy number of the transcripts of a given gene follows a lognormal-Poisson distribution. In the generalized linear mixed model, effects for line, time, line-by-time interaction, and plate used for Fluidigm PCR were treated as fixed effects, while individual animal was included as a random effect. Bayesian MCMC prior distributions for fixed effects and the random effect were derived by using expression data of GAPDH and RPL32 during the 24-h time course. The *p*-values associated with the effects of line, time and line-by-time interaction were adjusted by using the Benjamini-Hochberg (BH) method [39].

### RNA-sequencing

The 32 RNA samples used for the RT-qPCR assays above were also used for RNA-seq after depleting globin transcripts as described above. Library construction and sequencing were performed by the Beijing Genomics Institute (BGI, Hongkong, CN). Briefly, the RNA-seq libraries were constructed using the Illumina TruSeq RNA Sample Preparation Kit v2 (Illumina, San Diego, CA, USA) per the manufacturer’s instructions. Individual libraries were diluted to 2 nM and pooled in approximately equimolar amounts, with 8 libraries per pool. Paired-end sequencing (2 × 50 bases) was run on an Illumina Hiseq 2000 platform with one pool per lane.

### Quality control, preprocessing and alignment of RNA-seq reads

Read quality was checked and filtered by BGI using their custom scripts. For a pair of reads, the whole pair was removed if either read met the following criteria: (1) either read had more than 50% of their bases aligned to the adapter sequences; (2) either read contained more than 10% of ‘N’ bases; (3) either read had more than 40% of bases with PHRED+ 64 quality scores lower than 20. The kept reads were aligned to the pig reference genome *Sscrofa* 11.1 (version 90, Ensembl) using 2-pass *rna-STAR* (version 2.5.3a) with the ENCODE standard option settings plus two explicit option settings: -*-outFilterMismatchNoverReadLmax 0.1 --outFilterIntronMotifs RemoveNoncanonical* [40, 41]. The resulting BAM files were further processed by using *MMR* to assign multi-mapper reads to their most likely locations such that the variances of local basewise coverage were minimized [42]. Read counts per gene per library were summarized by using *featureCounts* (version 1.5.3) [43] with explicit settings *-d 30 -M*, with the pig genome GTF file (version 90, Ensembl) as the genomic annotation reference file. Prior to differential expression analysis, hemoglobin genes (HBA and HBB) and genes with few reads were removed from the count table, such that only genes with count per million (cpm) mapped reads greater than one in at least four samples were kept. This analysis resulted in a final count table for 12,703 genes. This count table was used for subsequent differential expression analysis and clustering analysis after further transformation and adjustment (see below).

### Differential expression analysis of RNA-seq data

Although in recent years, a few tools have been developed for time-course RNA-seq differential gene expression analysis [44, 45], there is no generally accepted, applicable method to analyze RNAseq data from a short time-series experiment, with less than five time points and a small sample size as this study, in which within-individual measures are generally correlated. Therefore we were unable to take into account expected within-animal correlation in our differential expression analysis. We used the R/Bioconductor package *DESeq2* (1.20.0) [46] for differential expression analysis for two reasons: (1) *DESeq2* adopts empirical Bayes shrinkage estimation for dispersions and fold changes, which improves stability and interpretability of estimates; (2) *DESeq2* allows statistical tests of differential expression with a specified minimum effect size, which avoids the issue that post hoc filtering of differentially expressed genes (DEGs) based on a fold change threshold results in a false discovery rate (FDR) that is not easy to interpret [46]. Additionally, known nuisance variables, such as RNA preparation batch, RIN, BW, and blood cell composition, could not well account for the treatment-unrelated variations of the complicated blood transcriptome. This is likely because several of them, such as the concentration of rare subtypes in peripheral blood (such as basophils, eosinophils, and monocytes), were not accurately measured. And more importantly, single cell RNA-seq data in human [47] and pig peripheral blood mononuclear cells (PBMCs) (Crystal L. Loving, Haibo Liu et al., unpublished) revealed that the lymphocytes are composed of transcriptionally very heterogeneous subtypes of cells. Surrogate variable analysis has been shown to be a powerful method to detect and adjust for hidden variations in high throughput gene expression data [48, 49]. Therefore, we decided to use surrogate variables estimated by the *svaseq* function of the R/Bioconductor package *sva* (v3.28.0) [50] to account for the hidden nuisance variations in our RNA-seq data. Six surrogate variables were estimated by using a full model that included terms for an intercept, line, time and line-by-time interaction, and a reduced model that included only an intercept term.

Differential expression analyses were conducted using *DESeq2* as mentioned above. Briefly, a generalized linear model was fitted for each gene in the count table, with a negative binomial response and a log link that included a *DEseq2* normalization offset and the effects of line, time and line-by-time interaction, and the six surrogate variables as estimated above. The *nbinomWaldTest* function was used to estimate and test the significance of regression coefficients with the following explicit parameter settings: *betaPrior = FALSE, maxit = 5000, useOptim = TRUE, useT = FALSE, useQR = TRUE*. Differentially expressed genes between conditions were identified by testing the significance of relevant contrasts and using the *results* function with the following explicit parameters: *alpha = 0.01, lfcThreshold = log2(1.2), altHypothesis = “greaterAbs”*, that is, testing whether the absolute values of the *log*_2_ fold changes between conditions were greater than *log*_*2*_(1.2). Thus, the estimates of the fold changes were shrunken by performing empirical Bayes shrinkage and their significances were tested by specifying a minimum effect size, which can improve the stability and interpretability of the estimates [46]. Multiple testing correction was performed by using the BH method [39]. Genes with absolute values of the *log*_2_ fold change greater than *log*_2_(1.2) and adjusted *p* values less than 0.05 were considered to be DEGs.

### Statistical analysis of body temperature, CBC and cytokine profile data

Cytokine levels below the lower limit of detection were replaced by one half of the smallest positive values in the cytokine dataset. The CBC data and the imputed cytokine data were natural *log*-transformed for further analyses. Rectal temperature (all 140 data points), the transformed CBC data (107 data points) and cytokine data (all 112 data points) were analyzed by using the SAS PROC MIXED procedure (SAS Institute Inc., Cary, NC) for repeated measures analysis. The models used for analyzing CBC data and rectal temperature data contained RFI line (Line), sampling time (Time), treatment (Treatment), two-way and three-way interactions of the three former factors, body weight (BW) and age (AgeOfChallenge) at LPS injection, and pen by rooms (PenRoom) as fixed effects. For cytokine data analysis, plate used in the assays (Plate) was also included as a fixed effect, along with all the independent variables used for rectal temperature data analysis. Six commonly used residual correlation structures for repeated measures data analysis, compound symmetry (CS), heterogeneous compound symmetry (CSH), autoregressive (AR), heterogeneous autoregressive (ARH), spatial power (SP), and unstructured (UN) correlation structures, were considered. For each response variable, the correlation structure giving the smallest Akaike information criterion (AIC) was selected for the final analysis. The goodness of fit of the models was assessed as descried [51]. Type III tests were performed for fixed effects and contrasts were adjusted using the Kenward-Roger method [52].

### Clustering of gene expression profiles

The filtered RNA-seq count data were transformed to *log* (*cpm*) by using the *voom* function of the *limma* package (v3.36.2) [53, 54]. The transformed gene expression levels were then adjusted for the nuisance variables, i.e.*,* the six surrogate variables used in the model for RNA-seq differential expression analysis. The adjusted *log (cpm)* for all individuals of the two lines were altogether used as input for the software STEM (v1.3.8), which is a tool specifically developed for short time-series expression data mining [55]. For parameter settings, see Supplementary Methods (Additional file 3). To test how likely the observed profiles were created at random, permutation tests were performed (For details, see Additional file 3). Expression profiles with more genes assigned than expected based on a null distribution derived from permutation, where gene expression values at 2, 6 and 24 hpi were randomly permuted within each animal for 50 iterations and used for STEM analysis (*q* < 0.05), were considered as significant profiles. Significant expression profiles were clustered together if the correlation coefficients of two mean profiles were no less than 0.6. GO term overrepresentation analysis and visualization were performed for genes in each cluster of profiles with a *q* value cutoff of 0.05 by using built-in functionality in STEM.

### Gene ontology term overrepresentation analysis (GOA)

Gene ontology (GO) annotation for pig genes was downloaded from Ensembl BioMart (Release 90). GO terms associated with less than 10 or more than 500 genes were excluded. For the RNA-seq data, 12,703 Ensembl Gene IDs with detectable expression in the blood samples were used as the background references. Hypergeometric tests of overrepresentation of GO terms by a gene list of interest were performed by using the *Cytoscape* (v3.4.0) [56] package *BiNGO* (v3.0.3) [57] with a *q* value cutoff of 0.05.

### Gene set enrichment analysis (GSEA)

Gene set enrichment analysis for the pig blood RNA-seq data was performed using the R/Bioconductor *gage* package (v2.22.0), which implements a gene permutation-based algorithm for gene set and pathway enrichment analysis [58]. Expression data adjusted for surrogate variables were used as inputs. The latest pig-specific KEGG pathways and GO terms-associated genes were downloaded from the KEGG database (Release 87.0) using the *gage* package [58] and Ensembl Biomart (Release 90) using the *biomaRt* package (v2.36.1) [59], respectively. The package *gage* was run in a paired-comparison mode when the samples compared were from the same individuals; otherwise it was run in a “group comparison” mode. Gene set enrichment analysis that made use of disregulation direction was performed for GO terms-derived gene sets, while for KEGG pathways-based gene sets, enrichment analysis both aware and unaware of dis-regulation direction were performed, with a *q* value cutoff of 0.05.

### Other statistical methods

A Sankey diagram showing status changes of DEGs over the time course of this study was generated by using the R package *alluvial* (v0.1–2, Bojanowski M. and Edwards R, unpublished). Surrogate variable-adjusted expression levels of differentially expressed genes or probesets were hierarchically clustered using the Ward.2 method with one minus the Pearson correlation coefficient as the distance measure to generate heatmaps using the *pheatmap* package (v1.0.10). 3D-principal component analysis (PCA) plots were generated using the R package *pca3d* (v0.10) (Weiner J. unpublished).

## Results

### Clinical data showed only slight differences in response to LPS between the two lines

**Fig. 2:**
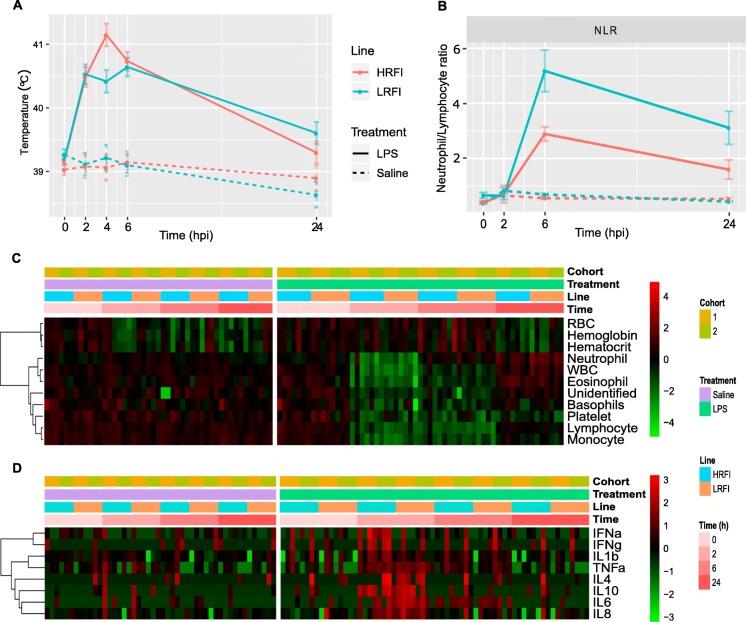
Intramuscular injection of LPS induced systemic inflammation. **a** LPS, but not saline, induced a fever response in pigs. The rectal temperature of pigs treated with LPS was significantly higher than baseline at 2, 4 and 6 hpi. Notably, the mean rectal temperature was significantly lower in the low-RFI animals than in the high-RFI animals at 4 hpi (adjusted *p <* 0.0075). At 6 hpi, the mean rectal temperature tended to be lower in the low-RFI animals than in the high-RFI lines, but this tendency reversed by 24 hpi. Shown are profiles of least square means of rectal temperature for the two lines at each time point after injection with LPS or saline. Error bars show standard errors of the means of rectal temperature of each line at each time point. **b** Neutrophil/lymphocyte ratios changed over the time course of the study in response to LPS stimulation. The neutrophil/lymphocyte ratio (NLR) was significantly higher at 6 and 24 hpi in the low-RFI animals than in the high-RFI lines treated with LPS (*p* < 0.03, two sided Mann-Whitney U test). **c** Dynamic CBC profiles in response to LPS or saline treatment showed LPS injection triggered inflammation in pigs of both lines, while saline did not. The quantity of each CBC parameter was standardized by normal transformation and displayed as a heatmap. **d** Cytokine dynamic profiles in response to LPS or saline injection in pigs. Cytokine concentrations were standardized by normal transformation and displayed as a heatmap. HRFI, high-RFI line; LRFI, low-RFI line

We intramuscularly injected pigs from the two lines divergently selected for RFI with LPS or saline to investigate whether divergent selection for RFI for multiple generations affects the pigs’ systemic inflammatory response over the 24 hpi. We did not detect a significant replicate difference in the rectal temperature profile (*p* > 0.1). So we performed joint analyses of the rectal temperature data from both replicates. As expected, the mean rectal temperature of pigs in the control group (*n* = 6 per line) only slightly fluctuated around 39 °C (39.07 ± 0.35 °C, mean ± standard deviation) during the time course of the study (Fig. 2a). However, the rectal temperature of LPS-treated pigs of both lines (*n* = 8 per line) at 2, 4 and 6 hpi was significantly higher than that at baseline (*p* < 9 × 10^− 11^). Slight differences in rectal temperature profiles were observed between the two lines. In detail, after LPS injection, the average rectal temperature of the high-RFI pigs increased by 1.31 °C at 2 hpi, peaked at 4 hpi, and then decreased and almost returned to baseline by 24 hpi. On the other hand, the average rectal temperature of the low-RFI pigs increased by 1.33 °C at 2 hpi, slightly decreased at 4 hpi, increased at 6 hpi, and then dropped towards baseline (Fig. 2a). By 24 hpi the average rectal temperature of the low-RFI pigs was still 0.4 °C higher than that at baseline (*p* = 0.034) (Fig. 2a). It is noteworthy that during the time course the maximal increase of the mean rectal temperature compared to their respective baseline temperature was 1.45 °C and 1.98 °C, for the low-RFI and high-RFI pigs treated with LPS, respectively. At 4 hpi, the rectal temperature of the high-RFI pigs was significantly higher than that of the low-RFI pigs (*p* = 0.0075). Additionally, the line-by-treatment interaction effect at 4 hpi was significant (*p* = 0.03). Detailed results of statistical analyses of the rectal temperature changes post treatment are shown in Additional file 4: Table S3. Pigs in the LPS treatment group had a rectal temperature higher than 40 °C from 2 to 6 hpi, indicating that intramuscular injection of LPS induced fever in pigs, which is a typical symptom of the systemic inflammation response.

Several parameters in the CBC profiles significantly changed in animals injected with LPS during the time course of the study, but no significant effects were detected for line, line-by-treatment, or line-by-time interaction effects (Fig. 2c, Additional file 5: Figure S1 and Additional file 6: Table S4). Median levels of red blood cells (RBCs), hemoglobin and hematocrit decreased at 2 hpi and remained lower than baseline at 24 hpi in the control group. For the treatment group, levels of red blood cells (RBCs), hemoglobin, and hematocrit decreased only at 2 hpi and 24 hpi (*p* < 0.02) in the low-RFI animals. In the high-RFI animals, levels of RBCs and hemoglobin slightly increased at 2 hpi, peaked at 6 hpi, and then decreased to levels lower than baseline at 24 hpi. Hemoglobin levels changed very similarly for both lines. The concentration of platelets did not significantly change in the control group, but continuously decreased post LPS injection in the treatment groups. In the control group, the concentration of each subtype of white blood cells (WBCs) did not significantly change relative to baseline regardless of line. For the LPS group, the concentration of each type of WBC significantly decreased at 2hpi, with the exception of basophils, which tended to be lower than baseline at 2 hpi, but was only significantly lower than baseline at 6 hpi. The level of neutrophils rebounded to baseline by 6 hpi and increased to a significantly higher level than baseline by 24 hpi, while the other subtypes of WBCs remained low (lymphocytes) or slightly increased (monocytes and eosinophils) at 6 hpi. By 24 hpi, the levels of basophils and eosinophils returned to baseline, while levels of lymphocytes and monocytes were still below baseline. The dynamic profile changes of WBCs in pigs treated with LPS suggests those pigs underwent leukopenia followed by neutrophilia. The neutrophil/lymphocyte ratio (NLR) peaked at 6 hpi, and was significantly different between the two lines at 6 and 24 hpi (*p* < 0.03, two-sided Mann-Whitney U test) (Fig. 2b). Summary statistics comparing the levels of the parameters of the CBC profiles in the peripheral blood post LPS injection in the control and treatment groups are shown in Additional file 6: Table S4. The dynamic profiles of WBCs during this time course further support the idea that systemic inflammation was induced by LPS in pigs.

Other common metrics of systemic inflammation are the levels of cytokines in the bloodstream. Plasma levels of eight cytokines (IL1β, IL4, IL6, IL8, IL10, TNFα, IFNα and IFNγ) at various time points following LPS injection are shown in Fig. 2d. Due to high variations in the measurements, which were also reported by Thorgersen et al. [60], no significant effects were detected for line, line-by-treatment or line-by-time interaction (Additional file 7: Table S5). However, animals treated with LPS tended to have higher cytokine levels in their bloodstream at 2 hpi compared to baseline, and compared to the control group at the equivalent time point. In the treatment group, the levels of three proinflammatory cytokines (IL6, IL1β and TNFα) were generally still higher than baseline at 6 hpi and returned to baseline by 24 hpi, while the levels of other cytokines nearly returned to baseline by 6 hpi. Summary statistics comparing the concentrations of the cytokines in the peripheral blood at 0, 2, 6 and 24 hpi in each experimental group are shown in Additional file 7: Table S5. The dynamic profiles of peripheral blood cytokines provide supportive evidence that LPS induced systemic inflammation in pigs.

### Targeted transcriptomic assays confirmed inflammatory response to LPS in blood but showed only slight differences between the two lines

**Fig. 3:**
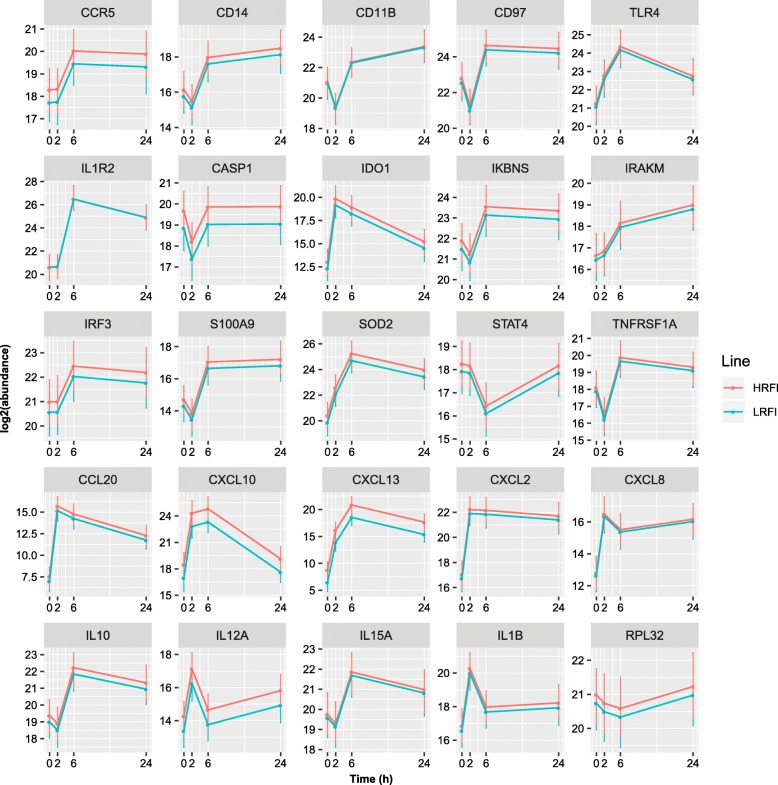
Expression profiles of inflammation-related genes as determined by RT-qPCR. Shown are the least square means of *log*_2_(abundance) ± 95% confidence intervals of 24 genes differentially expressed at least at one time point post LPS injection compared to baseline, and an assumed internal reference gene (RPL32). Notably, the expression level of the assumed internal reference was not stable over the time course of the study. For expression profiles of 36 selected and two assumed internal references, see Additional file 8: Figure S2. HRFI, high-RFI line; LRFI, low-RFI line

In addition to assessing levels of peripheral blood cytokines, changes in the transcript abundance for 36 inflammation-related genes were evaluated by RT-qPCR assays. The expression levels of 24 genes were significantly different from baseline for at least one time point after LPS injection (*q* < 0.05) (Fig. 3 and Additional file 8: Figure S2). No line-by-time interaction effects were detected for any gene assayed, while a line difference in gene expression was detected only for the CXCL13 gene (maximal *log*_2_(fold change) = 1.6, *p* = 0.004). Briefly, genes encoding nine cytokines/chemokines (IL1β, IL10, IL12A, IL15A, CXCL2, CXCL8, CXCL10, CXCL13, and CCL20), seven receptors (TLR4, CD11B, CD14, CD97, CCR5, TNFRSF1A and IL1R2), and eight TLR4/NFκB signaling pathway components or effectors (IRF3, IRAKM, IKBNS, STAT4, S100A9, SOD2, CASP1 and IDO1) were significantly up-regulated in peripheral blood after LPS injection relative to baseline for at least one time point (Fig. 3). The mRNA levels of proinflammatory cytokines and chemokines, including IL1β, IL12A, CXCL2, CXCL8, CXCL10 and CCL20, peaked at 2 hpi, while the mRNA amount of anti-inflammatory cytokines, IL1R2, TNFSF1A, and IL10, peaked at 6 hpi. The mRNA abundance for several genes involved in initiating the innate immune response, including TLR4, IRAK4, IRAKM, IRF3, NFκB1, RELA, and STAT1, and those involved in limiting the innate immune response, such as IKBNS, reached their peaks or nearly peaked by 6 hpi. Thus, the RT-qPCR data indicate that pigs injected with LPS had a typical inflammatory response, which progressed from an acute pro-inflammatory phase, to an anti-inflammatory phase and finally towards full resolution, very similar to the inflammatory response triggered by LPS in humans [24].

### Global mRNA profiling revealed only slight differences in response to LPS between the two lines

To gain a further understanding of the differential immune responses upon LPS exposure between the two lines, the peripheral blood transcriptomes of pigs from the two RFI lines (*n* = 4 per line) in the LPS treatment group at 0, 2, 6, and 24 hpi were profiled using RNA-seq. On average, 19.4 ± 2.5 million (mean ± standard deviation) read pairs per library were obtained and more than 92.2 ± 1.6% (mean ± standard deviation) of reads were uniquely mapped to the pig reference genome. After filtering out genes of very low expression, as well as hemoglobin genes (HBA and HBB), there were 12,703 genes whose expression levels met the minimal abundance requirement for downstream analyses. Principal component analysis suggested that samples from different time points were well-separated in the plane formed by the first and second principal components (PC1 and PC2), and samples from the two lines were well-separated in the PC3 direction (Additional file 9: Figure S3). Hierarchical clustering showed that samples at 24 hpi were closer to those at baseline than those at 2 and 6 hpi (Additional file 10: Figure S4).

**Fig. 4:**
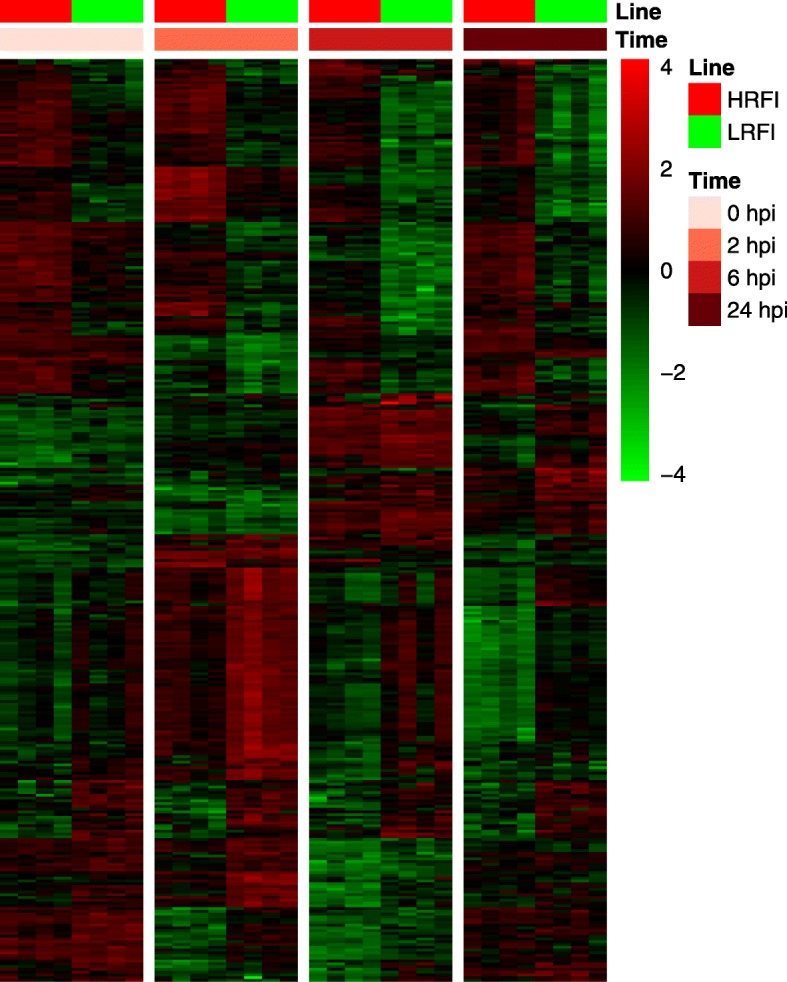
Heatmap showing expression profiles of DEGs between lines. Shown here are 334 gene that were differentially expressed (|*log*_2_(fold change)| ≥ *log*_2_(1.2) and *q* < 0.05) between the two lines at least at one time point during the time course of the study. HRFI, high-RFI line; LRFI, low-RFI line

**Fig. 5:**
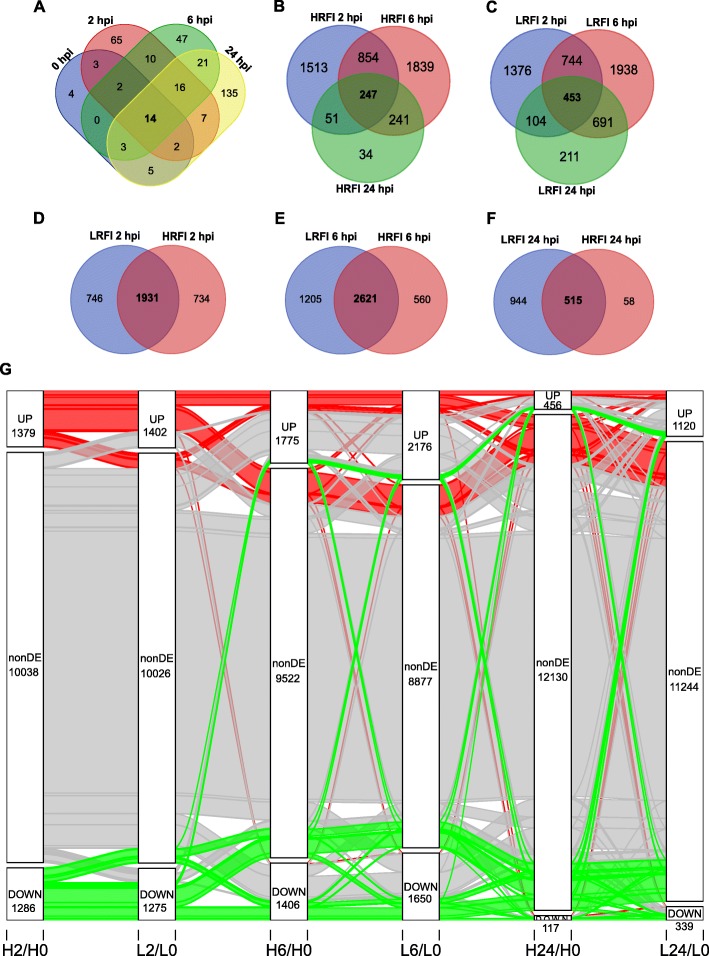
Venn and Sankey diagrams showing between-line, within-line DEGs (|*log*_2_(fold change)| ≥ *log*_2_(1.2) and *q* < 0.05) at different time points. **a** Venn diagram showing 334 DEGs between the two lines at each time point. **b** and **c** Venn diagrams showing DEGs between time points post LPS treatment and baseline in the high-RFI (**b**) and low-RFI (**c**) animals. **d**-**f** Venn diagrams showing the relationship of within-line DEGs at 2, 6, and 24 hpi relative to baseline, respectively, between low-RFI and high-RFI lines. HRFI, high-RFI line; LRFI, low-RFI line. **g** Sankey diagram showing dynamic differential expression of genes at 2, 6 and 24 hpi compared to baseline for both lines. Flow strips of up-regulated, non-significantly differentially expressed, and down-regulated genes at 2 hpi in the high-RFI animals compared to baseline are colored in red, gray and green, respectively. The number of genes in each differential expression categories at each time point for each line is shown. The heights of the strips and blocks are proportional to the gene counts. Labels on the horizontal axis are designated similarly. For example, H2/H0 means gene expression at 2 hpi compared to baseline for the high-RFI animals

We detected 33, 119, 113 and 203 genes that were differentially expressed between the two lines at 0, 2, 6 and 24 hpi, respectively (*q* < 0.05, Additional file 11: Table S6). Overall, we detected 334 unique genes which were differentially expressed between the two lines for at least one time point during the time course of the study. A heatmap showing expression profiles of these 334 DEGs is shown in Fig. 4. Fourteen of these 334 genes were differentially expressed between the two lines at all four time points studied, as shown by the Venn diagram (Fig. 5a).

**Fig. 6:**
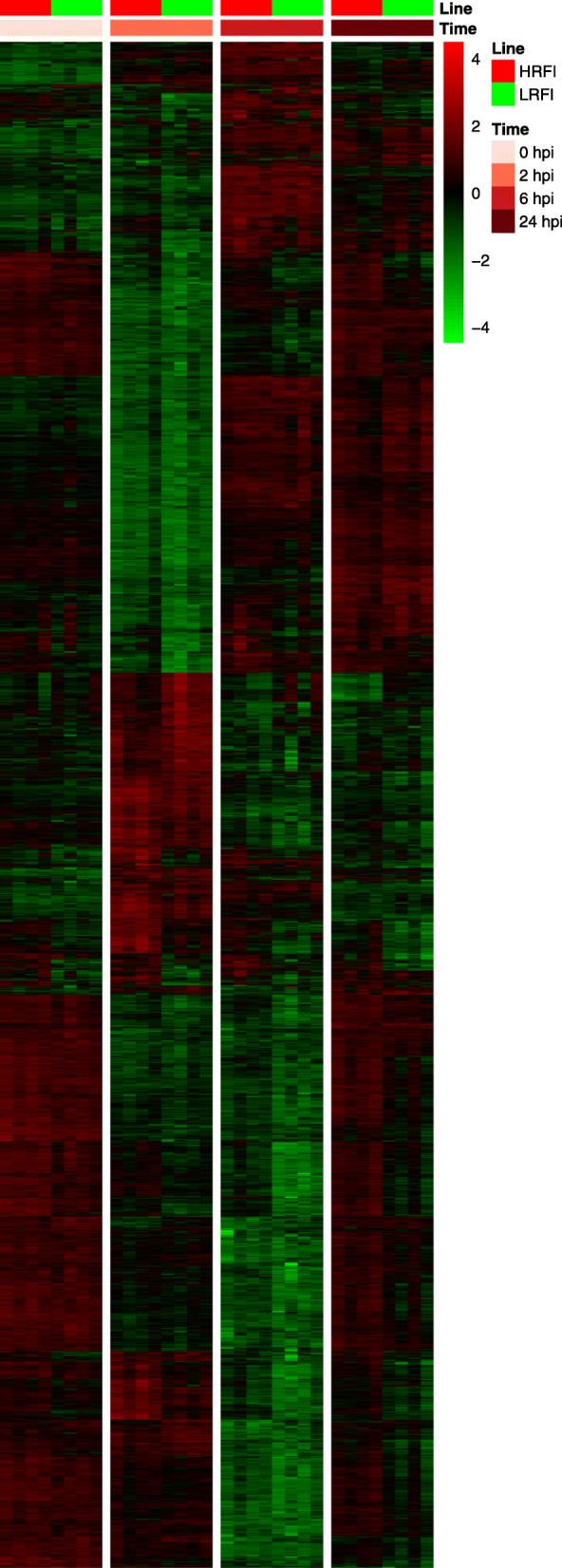
Heatmap showing expression profiles of DEGs that were responsive to LPS stimulation. Shown are 6296 genes that were differentially expressed (|*log*_2_(fold change)| ≥ *log*_2_(1.2) and *q* < 0.05) at least at one time point post LPS treatment relative to baseline for each line. HRFI, high-RFI line; LRFI, low-RFI line

To find genes whose expression significantly changed after LPS injection, we compared gene expression between time points after injection and at baseline for each line and identified a large number of DEGs (Fig. 6 and Additional file 12: Table S7). DEGs in peripheral blood cells of the two lines at the given time points relative to baseline largely overlapped but DEGs of the two lines at different time points only moderately overlapped (Fig. 5b-g). In total, 6296 unique genes were differentially expressed in at least one line for at least one time point post LPS injection relative to baseline. Significant line-by-time interaction effects were only detected for two genes (AQP2 and CXCL11), which suggests that the line-by-time interaction effects were negligible. A full list of within-line DEGs at 2, 6, and 24 hpi compared to baseline is available in Additional file 12: Table S7. Genes that were differentially expressed at least for one time point post LPS injection compared to baseline in at least one line were hierarchically clustered and are displayed as a heatmap (Fig. 6). Expression patterns of gene sets derived from GO biological process terms, GO:0006954 (inflammatory response), GO:0002526 (acute inflammatory response) and GO:0032496 (response to LPS), are shown in Additional file 13: Figure S5.

**Fig. 7:**
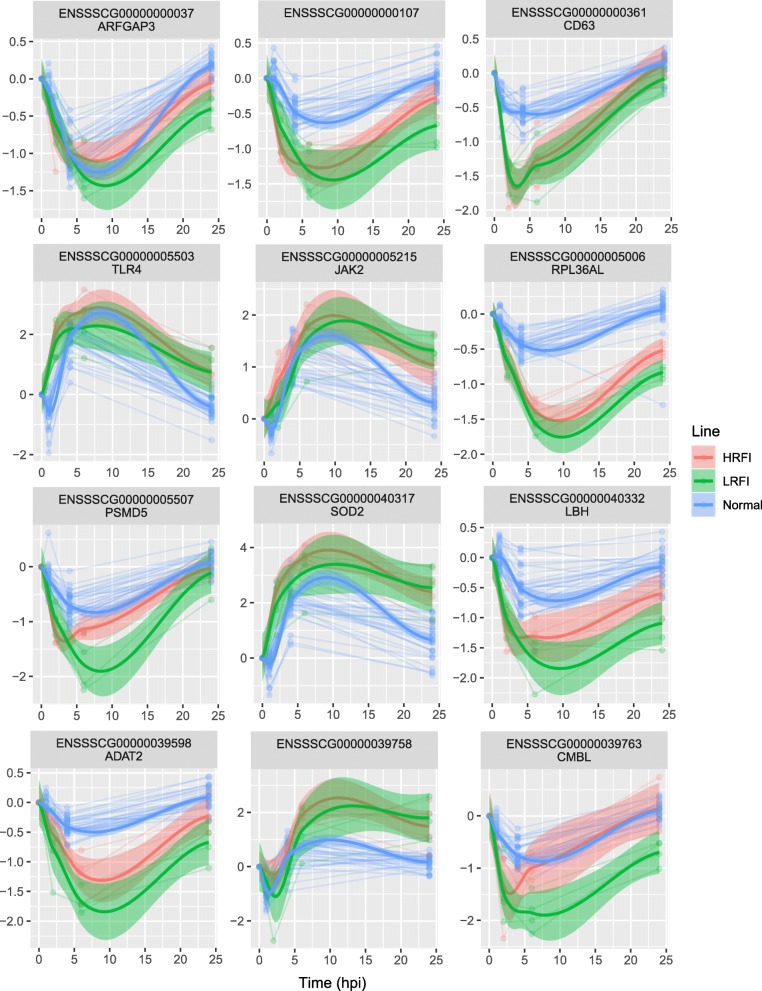
Cross-validated representative profiles of genes that were responsive to LPS stimulation. Genes showing differential expression (|*log*_2_(fold change)| ≥ *log*_2_(1.2) and *q* < 0.05) post LPS injection compared to baseline were cross-validated by using independent time-series gene expression microarray data on response of pigs’ whole blood to LPS [23]. The y-axis shows the *log*_*2*_ fold change of gene expression at each time point relative to baseline, estimated by *DESeq2* or *limma*. Smoothed expression profiles of individual genes per line were inferred by using LOWESS (Locally Weighted Scatterplot Smoothing). HRFI, high-RFI line; LRFI, low-RFI line; Normal, pigs not selected for RFI [23]

The 6296 DEGs included 23 of the 24 genes that were detected as differentially expressed by the RT-qPCR assays for at least one time point post injection relative to baseline. Notably, 1610 of the 6296 DEGs were cross-validated by another gene expression microarray experiment that profiled the longitudinal whole blood transcriptomic response to LPS [23] (For reanalysis methods, see Additional file 3). Some of the 1610 cross-validated genes showed very similar dynamic patterns in both studies, while others showed delayed response patterns in Terenina et al. [23] compared to our study. Dynamic expression patterns of some representative cross-validated genes are shown in Fig. 7 and Additional file 14: Figure S6.

In summary, differential gene expression analyses suggested the pigs’ blood cell transcriptomes were dramatically altered by LPS injection in both lines in largely similar ways, although expression dynamics of some genes were slightly different between the two lines.

### Functional annotation of between-line transcriptomic differences revealed slight differences in dynamics of responses to LPS between the two lines

The DEGs between the two lines at each time point were first functionally annotated by using GOA (Additional file 15: Table S8). Among DEGs between the two lines at baseline, overrepresented were genes with transmembrane transporter activity (GO:0022857), and more specifically, chloride channel activity. No GO terms were significantly overrepresented among the list of DEGs between the two lines at 2 hpi. Genes involved in porphyrin/heme biosynthesis and cellular iron ion homeostasis were overrepresented among the between-line DEGs at 6 hpi. At 24 hpi, genes involved in porphyrin/heme biosynthesis, as well as the immune response (GO:0006955), and more specifically, genes involved in antigen processing and presentation of peptide or polysaccharide antigen via MHC class II (GO:0002504), were overrepresented among the between-line DEGs. The expression levels of six porcine MHC class II genes were significantly lower in the low-RFI line than in the high-RFI line at 24 hpi (Additional file 11: Table S6 and Additional file 16: Figure S7).

**Fig. 8:**
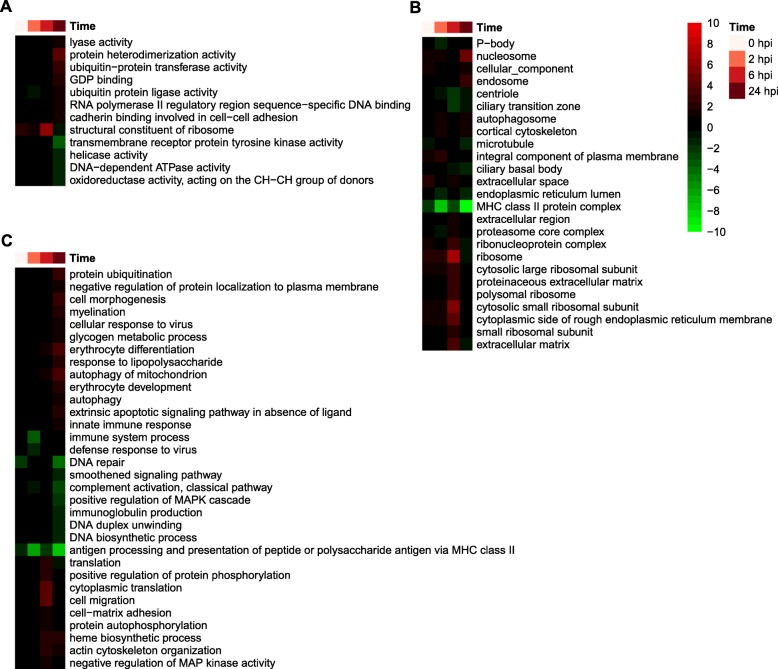
GO term-derived gene sets enriched among DEGs between the two lines at each time point. Shown are gene sets significantly (*q* < 0.05) enriched at at least one time point. **a**-**c** Enriched gene sets derived from GO molecular functions (**a**), cellular components (**b**), and biological processes (**c**), respectively. Values displayed as heatmaps are *log*_*10*_(*q* value) or - *log*_*10*_(*q* value), respectively, if gene sets were enriched among genes of lower or higher expression levels in the low-RFI animals than in the high-RFI animals at a given time point. HRFI, high-RFI line; LRFI, low-RFI line

**Fig. 9:**
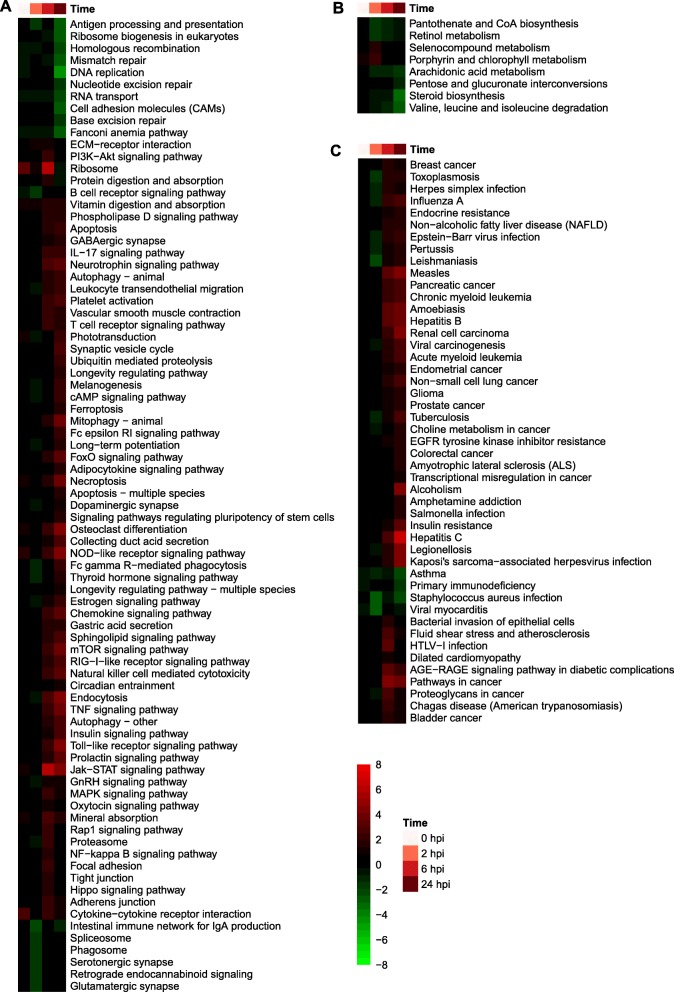
KEGG pathway-derived gene sets enriched among DEGs between the two lines at each time point. Shown are gene sets significantly (*q* < 0.01) enriched at at least one time point. **a**-**c** Enriched gene sets derived from KEGG signaling pathways (**a**), metabolic pathways (**b**), and disease pathways (**c**), respectively. Values displayed as heatmaps are *log*_*10*_(*q* value) or -*log*_*10*_(*q* value), respectively, if gene sets were enriched among genes of lower or higher expression levels in the low-RFI animals than in the high-RFI animals at a given time point. HRFI, high-RFI line; LRFI, low-RFI line

The functions of the DEGs between the two lines at each time point were then analyzed using GSEA, which is more sensitive than GOA, especially when the transcriptional differences between conditions are small [61]. Gene set enrichment analysis based on comparing mean expression levels of genes in the low-RFI versus the high-RFI line not only largely recapitulated the results of GOA, but also indicated many more gene sets of interest were enriched (Figs. 8 and 9). For example, the gene sets for antigen processing and presentation of peptide or polysaccharide antigen via MHC class II were generally enriched among genes that had lower expression levels in the low-RFI line than in the high-RFI pigs, especially at 2 and 24 hpi (Fig. 8). Furthermore, expression levels of genes associated with translation, cytoplasmic translation, and ribosome were higher in the low-RFI line than in the high-RFI animals at 6 hpi, but lower at 24 hpi (Fig. 8). Expression levels of genes associated with protein ubiquitination, cellular response to virus, glycogen metabolic process, erythrocyte differentiation, response to LPS, autophagy, innate immune response, cell migration, and actin cytoskeleton organization were higher in the low-RFI animals than in the high-RFI animals at 24 hpi. However, expression levels of genes associated with immune system process, defense response to virus, DNA repair, smoothened signaling pathway, complement activation via classical pathway, positive regulation of MAPK cascade, IgG production, and DNA biosynthesis were lower in the low-RFI animals than in the high-RFI animals at 0, 2 and/or 24 hpi (Fig. 8).

Results of GSEA based on gene sets derived from KEGG pathways were largely consistent with those of GSEA based on gene sets derived from GO terms, but provided information on the fine differences between the two lines in terms of the dynamics of their inflammatory responses to LPS (Fig. 9). Expression levels of genes associated with antigen processing and presentation, ribosome biogenesis, DNA repair and homologous recombination, RNA transport, cell adhesion, and the Fanconi anemia pathway were lower in the low-RFI animals than in the high-RFI animals, especially at 24 hpi. Genes involved in the B cell receptor signaling pathway, spliceosome, phagosome, serotonergic synapse, glutamatergic synapse and retrograde endocannabinoid signaling had lower expression levels in the low-RFI animals than in the high-RFI animals at 2 hpi. Gene sets for ribosome and cytokine-cytokine receptor interaction pathways were enriched among genes that had higher expression levels in the low-RFI animals than in the high-RFI animals at baseline and at 6 hpi. Gene sets for leukocyte transendothelial migration, phototransduction, cAMP signaling pathway, dopaminergic synapse, endocytosis, thyroid hormone signaling pathway, estrogen signaling pathway, GnRH signaling pathway and proteasome were enriched among genes with lower expression levels in the low-RFI animals than in the high-RFI animals at 2 hpi, but were enriched among genes that had higher expression levels in the low-RFI animals than in the high-RFI animals at 6 and/or 24 hpi. Gene sets for IL-17 signaling pathway, autophagy, mitophagy, T cell receptor signaling pathway, apoptosis, necrosis, NOD-like receptor signaling pathway, RIG-I-like receptor signaling pathway, chemokine signaling pathway, TNF signaling, toll-like receptor signaling pathway, Jak-STAT signaling pathway, and NFκB signaling pathway were enriched among genes that had higher expression levels in the low-RFI animals than in the high-RFI animals at 6 and/or 24 hpi. Gene sets for pantothenate and CoA biosynthesis, retinol metabolism pathway, arachidonic acid metabolism, steroid biosynthesis, and valine, leucine and isoleucine degradation were enriched among genes showing lower expression in the low-RFI animals than in the high-RFI animals post LPS injection. Gene sets for multiple KEGG disease pathways were enriched among genes having lower expression levels in the low-RFI animals than in the high-RFI animals at 2 hpi, but these disease pathways were enriched among genes that had higher expression levels in the low-RFI animals than in the high-RFI animals from 6 to 24 hpi. KEGG pathways-based GSEA suggested that the low-RFI animals had a lower-level inflammatory response than the high-RFI animals at 2 hpi, but a higher-level inflammatory response at 6 and 24 hpi.

### Functional comparison of within-line transcriptional responses to LPS revealed further minor differences between the two lines

Since the two lines showed very similar expression profiles at baseline, we also carried out GSEA by comparing gene expression at 2, 6 and 24 hpi to baseline for each line separately. We then compared the enrichment level of each set between the two lines. These analyses are supposed to be more powerful than the enrichment analysis mentioned above because within-individual correlations in gene expression were considered [58].

**Fig. 10:**
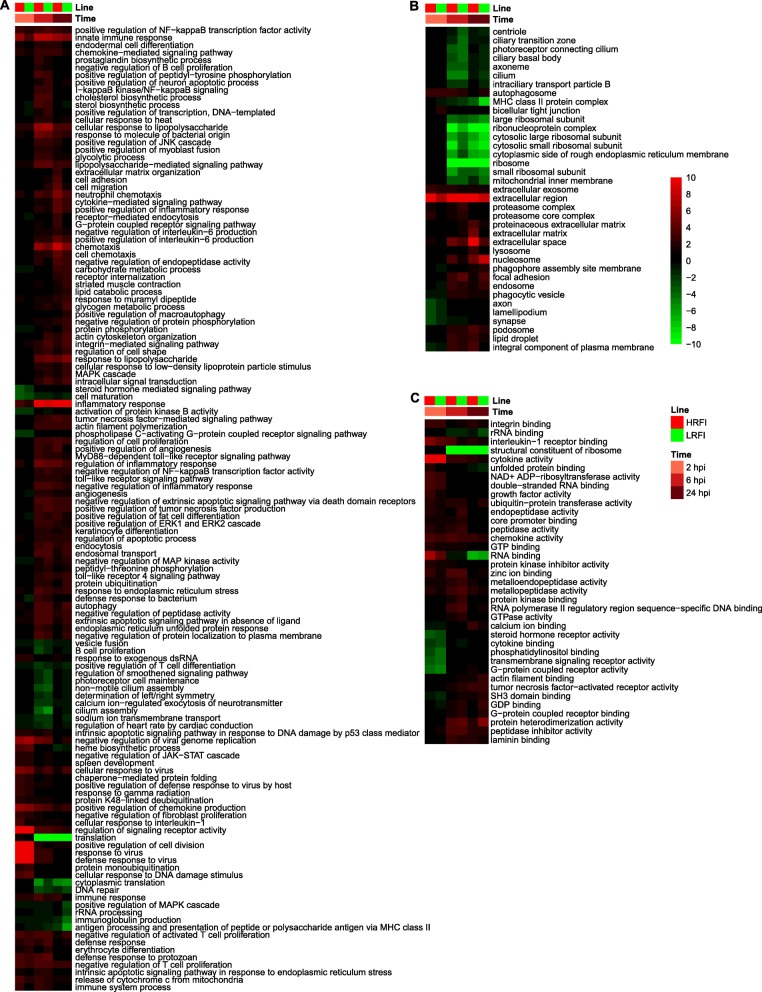
GO term-derived gene sets enriched among DEGs post LPS injection relative to baseline. Shown are gene sets significantly (*q* < 0.01) enriched at at least one time point. **a**-**c** Enriched gene sets derived from GO biological processes (**a**), cellular components (**b**), and molecular functions (**c**), respectively. Values displayed as heatmaps are *log*_*10*_(*q* value) or - *log*_*10*_(*q* value), respectively, if gene sets were enriched among genes of lower or higher expression levels in the low-RFI animals than in the high-RFI animals at a given time point. For better visualization, values greater than 10 or less than − 10 were set to 10 or − 10, respectively. HRFI, high-RFI line; LRFI, low-RFI line

Results of this alternative analysis reiterated most of the differences suggested by functional analyses of between-line transcriptome differences. Here we only mention some of the additional differences between the two lines that were identified by this alternative analysis. GO terms-derived gene sets, which were significantly enriched among genes that changed expression levels post LPS injection, are shown in Fig. 10. Gene sets for innate immune response were less enriched among genes that had higher expression levels in the low-RFI line than in the high-RFI line at 2 and 24 hpi compared to baseline. Gene sets for neutrophil chemotaxis and regulation of inflammatory response were less enriched among genes that had higher expression levels at 2, 6 and 24 hpi compared to baseline in the low-RFI line than in the high-RFI line. Gene sets for response to molecule of bacterial origin, chemotaxis, positive regulation of inflammatory response, negative regulation of endopeptidase activity, and positive regulation of IL-6 production were less enriched among genes that had higher expression levels at 6 and 24 hpi in the low-RFI line than in the high-RFI line. The gene set for cilium assembly and B cell proliferation were more and less, respectively, enriched among genes that had lower expression levels at 6 and 24 hpi compared to baseline in the low-RFI line than in the high-RFI line. Gene sets for prostaglandin biosynthesis were less enriched among genes that had higher expression levels at 6 hpi compared to baseline in the low-RFI line than in the high-RFI line. Gene sets for sterol biosynthesis were less enriched among genes that had higher expression levels at 6 hpi compared to baseline in the low-RFI line than in the high-RFI line, but more enriched among genes that had lower expression levels at 2 and 24 hpi compared to baseline in the low-RFI line than in the high-RFI line. As for metabolic differences, gene sets for lipid catabolism and glycogen metabolic process were more enriched among genes that had higher expression levels at 24 hpi, and 6 and 24 hpi, respectively, compared to baseline in the low-RFI line than in the high-RFI line.

KEGG signaling, metabolism and disease pathways that were significantly enriched among genes that were responsive to LPS stimulation for each line are shown in Additional file 17: Figure S8. Several additional enriched gene sets that were unique to KEGG pathways-based alternative analysis included the gene sets for the neurotrophin signaling, cAMP signaling, insulin signaling, platelet activation, and sphingolipid signaling, and the prolactin signaling pathways. These gene sets were more enriched among genes of higher expression level at 6 and 24 hpi compared to baseline in the low-RFI line than in the high-RFI line. Gene sets for fatty acid elongation and butanoate metabolism were more enriched among genes of lower expression levels at 6 and 24 hpi compared to baseline in the low-RFI animals than in the high-RFI pigs. Gene sets for five signaling pathways that are closely related to inflammation, i.e. the RIG-I-like receptor signaling, necroptosis, TNF signaling, Jak-STAT signaling, Toll-like receptor signaling, and the NOD-like receptor signaling, were less enriched among genes that had higher expression levels at 2 hpi compared to baseline in the low-RFI line than in the high-RFI line, but more enriched among genes that had higher expression levels at 6 and 24 hpi compared to baseline in the low-RFI line than in the high-RFI line. Along with results based on gene sets derived from KEGG disease pathways, the differential enrichment between the two lines suggests the low-RFI line had a lower-level inflammatory response than the high-RFI line at 2 hpi, but a higher-level inflammatory response at 6 and 24 hpi.

### Genes co-expressed in response to LPS stimulation showed enriched biological functions related to inflammatory response

**Fig. 11:**
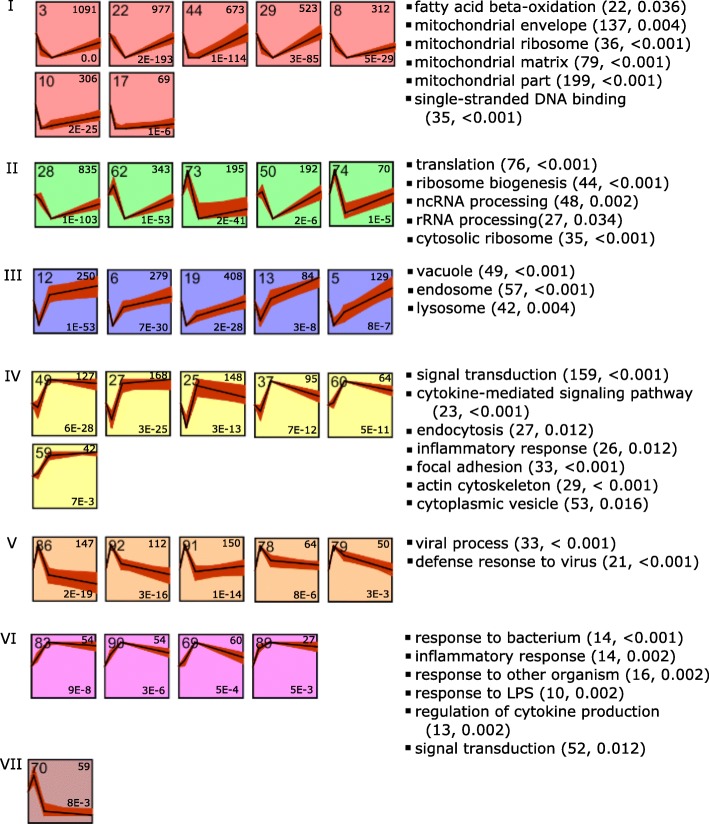
Clusters of significant expression profiles of genes responsive to LPS stimulation. Thirty-three significant profiles were clustered into seven clusters (shown on the left) based on profile similarities (correlation cutoff ≥0.6) by using STEM. Profiles of the same color belong to the same cluster. Profile IDs, sizes, and significance (*p* values) are shown on the upper left, upper right and lower right corners of individual profile plots, respectively. Significantly overrepresented GO terms by each cluster are displayed on the right. The number of genes and *q* values associated with a significant term are displayed in the parentheses

Lastly, gene co-expression analysis was performed to complement the conventional differential expression analysis, which considered each gene independently, and the GSEA, which considered groups of genes with known related functions together. Given that only a few significant line-by-time interactions were detected, we performed a clustering analysis of the gene expression profiles of both lines jointly to improve the stability of the clustered profiles. Thirty-three significant expression profiles were identified and these were further merged into seven clusters based on profile similarity (Fig. 11). Main GO terms that were overrepresented among genes in each cluster are shown in Fig. 11. Overrepresented GO terms among genes in Cluster I included fatty acid beta-oxidation, mitochondrial part, and mitochondrial ribosome. Translation and ribosome biogenesis were overrepresented among genes of cluster II. Genes functioning in vacuoles, endosomes, and lysosomes were overrepresented among cluster III genes. GO terms including signal transduction, endocytosis, and inflammatory response were overrepresented among genes in cluster IV. Genes involved in defense response to viruses and bacteria were overrepresented among clusters V and VI.

## Discussion

Typically, LPS exposure in pigs can induce a febrile response, hemodynamics, increased secretion of inflammatory cytokines, tissue-specific protein translation inhibition, and drastically changed metabolism [30, 32, 62–68]. The febrile response is believed to be an adaptive response to infection, which can contribute to controlling proliferation of invading pathogens in hosts [69, 70]. It is usually manifested as an increase in body temperature, typically by 0.5 to 4 °C [71]. In order for the host to maintain an elevated body temperature, it has been estimated that for every 1 °C increase in febrile core body temperature, the metabolic rate of the body would increase by approximately 13% [72]. Therefore, it is reasonable to expect that the different ways pigs perceive and respond to inflammatory agonists, such as LPS, can contribute to differences in feed efficiency between animals. To assess this, pigs from two lines that had been divergently selected for RFI for eight generations, were injected with LPS to assess differential immune activation that may have resulted from genetic selection on feed efficiency.

The LPS treatment induced a systemic inflammatory response in both lines, as evidenced by fever, and the typical dynamic changes in WBC levels and plasma cytokines. The change in body temperature of pigs from the high-RFI line and the dynamics of the CBC profiles of both lines post LPS treatment were similar to those previously reported [23, 60, 73, 74]. Interestingly, the body temperature of the low-RFI pigs at 4 hpi was significantly lower than that of the high-RFI pigs, which suggests that the low-RFI animals initiated a lower-level inflammatory response upon systemic LPS exposure. Consistent with this result, the plasma levels of two proinflammatory cytokines (IL1β and IFNγ) tended to be lower in the low-RFI line than in the high-RFI line at 2 hpi, although the plasma levels of both cytokines increased following LPS injection in both lines. The NLR is a marker that can be used to detect an ongoing systemic inflammatory response [75] and has been shown to increase in patients with cancer and psychiatric disorders, such as breast cancer [76] and Alzheimer’s disease [77], both of which have an inflammation component. In this study, the NLR in the low-RFI line was not different from that in the high-RFI line at either baseline or 2 hpi, but the NLR was higher in the low-RFI animals at both 6 and 24 hpi compared to the high-RFI line. A similar change in NLR during LPS stimulation was reported by Kvidera et al. [62]. The dynamic changes in NLR and rectal temperature suggested that the low-RFI line initiated a slightly lower-level inflammatory response than the high-RFI line, but the inflammation was resolved slightly more slowly in the low-RFI line than in the high-RFI line.

By global gene expression profiling of peripheral blood cells before and after LPS injection, only a small number of DEGs between the two lines were identified at each time point. However, slight differences in the biological processes and pathways between the two lines in response to LPS exposure were detected using more powerful and sensitive functional annotation tools. Functional analysis of the small number of between-line DEGs at each time point by GOA suggest that some biological processes that occurred post LPS injection were different between the two lines, including heme biosynthesis and antigen processing and presentation of peptide or polysaccharide antigen via MHC class II. GSEA of gene expression differences between the two lines at each time point reiterated and extended the findings from the GOA. These functional analyses suggested that compared to baseline, expression levels of genes related to heme biosynthesis were up-regulated at 2 hpi, returned to baseline at 6 hpi, and were further down-regulated at 24 hpi. However, the extent to which these genes were down-regulated after 2 hpi was smaller in the low-RFI animals than in the high-RFI pigs. Heme is a multi-functional, ubiquitously expressed essential molecule in higher animals [78]. As a component of hemoglobin, it facilitates gas exchange by binding oxygen and CO_2_. Notably, the inflammatory process is associated with an increased oxygen demand [32]. Thus, increased heme biosynthesis might help alleviate the increased oxygen demand, allowing the low-RFI animals to better handle hypoxia induced by an inflammatory response. Interestingly, Jégou et al. [12] reported that the concentration of RBCs at a young age was significantly higher in the INRA low-RFI line than in the high-RFI line. We also observed that the average concentration of RBCs and hemoglobin tended to be higher in the low-RFI pigs than in the high-RFI pigs at baseline, and at 6 and 24 hpi (Additional file 5: Figure S1), which was consistent with another study on the ISU RFI lines [10]. On the other hand, it is also known that LPS can induce hemolysis [79], which leads to a release of hemoglobin and free heme from RBCs. Notably, free heme is cytotoxic and can enhance the generation of reactive oxygen species (ROS), inflammation, and apoptosis by activating specific receptors, including the TLR4 signaling pathway [80]. It would be interesting to investigate the concentration of free heme in blood in the two lines, since the low-RFI animals might have slightly higher levels of heme biosynthesis and potentially higher levels of free heme in circulation.

Both GSEA and GOA also suggested that the low-RFI line differed from the high-RFI line in antigen processing and presentation via MHC class II, especially at 2 and 24 hpi. Expression levels of six MHC class II genes were significantly lower in the low-RFI line compared to the high-RFI line at 24 hpi (Additional file 16: Figure S7). Thus, blood cells in the low-RFI line might process and present exogenous antigens via MHC class II at a reduced capacity when compared to the cells in high-RFI line during the resolving phase of the systemic inflammatory response. This observation is in line with findings from previous in vitro studies of PBMC responses to LPS stimulation in pigs and rabbits [27, 81]. The physiological effects of lower expression levels of MHC II genes after LPS stimulation in the low-RFI line compared to the high-RFI line is unclear, as is its potential contribution to differences in feed efficiency between the two lines. However, evidence from both an experimental dual challenge of these ISU RFI lines with *Mycoplasma hyopneumoniae* and *Lawsonia intracellularis* and a study where the INRA RFI lines were exposed to less unsanitary housing conditions suggests that low-RFI pigs perform as well as, or even better than, high-RFI pigs under such immune challenges [82, 83]. Therefore, the lower expression levels of MHC class II genes in the low-RFI animals compared with the high-RFI animals might not be detrimental during health challenges.

Tissue-specific translational inhibition in response to infection is widely observed in multiple species, from invertebrates to mammals [84]. In rodents and pigs, it has been shown that LPS stimulation reduces skeletal muscle protein synthesis by modulating activities and availability of translation initiation factors through the mTOR pathway [64, 65, 85–87]. Here, we found genes that encode components of the ribosome and other translational machinery, including translation initiation factors and translation elongation factors, were down-regulated in response to LPS stimulation at 6 and 24 hpi in both lines. Notably, gene sets for ribosome and translation machinery were more enriched in down-regulated genes in the high-RFI line than in the low-RFI line at 6 hpi. In contrast, at 24 hpi, they were more enriched in down-regulated genes in the low-RFI line than in the high-RFI line. The expression levels of ribosomal protein-encoding genes were higher in the low-RFI animals than in the high-RFI animals at baseline, and at 2 and 6 hpi (Fig. 8), but lower in the low-RFI animals than in the high-RFI animals at 24 hpi. These results suggest that protein synthesis might be less suppressed in the low-RFI animals than in the high-RFI animals post LPS injection until some point between 6 and 24 hpi. Consistent with these results, the basal expression level of genes involved in translation elongation were higher in multiple tissues, including whole blood, in low-RFI pigs compared to high-RFI pigs [12, 13]. It is unclear whether translation inhibition is a true direct sensor of bacterial pathogens or an indicator of a more general host metabolic stress response to LPS [84], although a recent study reported that translational inhibition plays an important role in negative feedback regulation of the inflammatory response in macrophages [88]. However, if protein translation machinery in skeletal muscle is also less inhibited in the low-RFI pigs than in the high-RFI pigs as seen in the peripheral blood during LPS stimulation, this will at least partially explain why the low-RFI line is more feed-efficient than the high-RFI line.

Both clinical and global transcriptomic data suggested that these two lines divergently selected for RFI had a similar systemic inflammatory response triggered by LPS injection. However, the low-RFI line initiated a less striking but longer-lasting inflammatory response post LPS stimulation than the high-RFI line. Three lines of previous evidence support our results: (1) low-RFI animals had lower basal levels of serum acute phase protein (haptoglobin), an indication of lower basal inflammation, than high-RFI animals from the seventh generation of the same selected lines as used in this study [89]; (2) blood expression levels of genes involved in defense responses, as well as inflammatory and immune responses, were lower in the low-RFI pigs than in the high-RFI pigs [12]; and (3) expression levels of genes that participate in immune response, cytokine production and defense responses were lower in multiple tissues in the INRA low-RFI pigs than in the high-RFI pigs [13]. A slightly reduced inflammatory response might reduce the energy used during this response, as well as restrict self-damage due to inflammation. Such genetic differences may be beneficial for pig production if the existing level of inflammation is enough for the host to remove pathogens and recover from infection. Furthermore, as mentioned previously, both a 21-day longitudinal study where the two ISU RFI lines were dually challenged with *M. hyopneumoniae* and *L. intracellularis* and another study where the INRA RFI lines were housed in a less sanitary environment indicated that low-RFI animals performed as well as, or even better than, high-RFI animals under real-life health challenge conditions [82, 83]. Thus, it is reasonable to assume that the slightly lower level of inflammatory response in the low-RFI line than in the high-RFI line is not detrimental, if there is any side effect.

Finally, we acknowledge the limitations of our RNA-seq differential expression analysis and GOA since we did not consider the within-animal correlation of gene expression between time points. Our resulting DEG lists may not be complete or may contain false positives, which could affect GOA results that are based on DEG lists. However, the overall conclusions would not be seriously affected for the following reasons: 1) all the clinical and RT-qPCR data, which were consistent with the transcriptomic data, were analyzed using methods that properly considered within-animal correlations between time points; 2) in addition to DESeq2-based differential expression analysis and the GOA, we performed GSEA and STEM-based profile analysis, which accounted for the within-animal correlations and the results from these two analyses were largely consistent with the results from the former two analyses.

## Conclusions

Pigs divergently selected for RFI responded to LPS exposure with a systemic inflammatory response that was largely similar between the low-RFI and high-RFI lines. However, the low-RFI animals had a lower level of inflammation initially and took slightly longer to resolve the inflammation than the high-RFI animals. Our work indicates that selection for feed efficient based on RFI did not significantly compromise the pig’s capability to respond to an acute systemic inflammatory trigger.

## Supplementary information

**Additional file 1: Table S1.** Metadata for RNA-seq samples. (XLSX 30 kb)

**Additional file 2: Table S2.** Primers used to confirm inflammatory response in pigs stimulated with LPS by RT-qPCR. (XLSX 17 kb)

**Additional file 3:** Supplementary methods. This file contains parameter settings for STEM analyses. (DOCX 27 kb)

**Additional file 4: Table S3.** Summary of statistical analysis of rectal temperature data. (XLSX 13 kb)

**Additional file 5: Figure S1.** Profiles of CBC parameters during the 24-h time course. Shown are medians of measurement of each CBC parameter ± median absolute deviation (MAD). The units for the y-axes are 10^6^/μl (RBCs), % (Hematocrit), g/dl (Hemoglobin), 10^3^/μl (Platelet), 10^3^/μl (WBCs), 10^3^/μl (Lymphocytes, Neutrophils, Monocytes, Basophils and Eosinophils), pg (MCH), g/dl (MCHC), fl (MCV, MPV) and % (RDW). HRFI, high-RFI line; LRFI, low-RFI line. (PDF 17 kb)

**Additional file 6: Table S4.** Summary of statistical analysis of CBC data. (XLSX 38 kb)

**Additional file 7: Table S5.** Summary of statistical analysis of plasma cytokine data. (XLSX 24 kb)

**Additional file 8: Figure S2.** Expression profiles of inflammation-related genes determined by RT-qPCR. Shown are least square means of *log*_2_(abundance) ± 95% confidence intervals of 36 genes, and two assumed internal reference genes (RPL32 and GAPDH). Notably, the expression levels of the assumed internal references were not stable over the time course of the study. HRFI, high-RFI line; LRFI, low-RFI line. (PDF 144 kb)

**Additional file 9: Figure S3.** 3D-PCA plots showing the relationship of RNA-seq samples. H, high-RFI line; L, low-RFI line; Tx, x hpi. H_T0, samples at baseline from the high-RFI line treated with LPS. (GIF 2332 kb)

**Additional file 10: Figure S4.** Heatmap showing sample similarities. Euclidian distances between samples were calculated based on adjusted *log*_2_*(cpm)* gene expression. Samples were then hierarchically clustered using the complete linkage clustering method. *Pheatmap* was used to generate the heatmap. H, high-RFI line; L, low-RFI line; Tx, x hpi. H_LPS_T2_P41, 2 hpi sample of a high-RFI pig with ear tag 41treated with LPS. (PDF 14 kb)

**Additional file 11: Table S6.** Between-line DEGs at each time point. The identifiers of the 14 genes persistently differentially expressed between the two lines are marked with red color. (XLSX 5264 kb)

**Additional file 12: Table S7.** Within-line DEGs post LPS injection compared to baseline. (XLSX 8237 kb)

**Additional file 13: Figure S5.** Expression patterns of gene sets derived from GO-BP terms directly related to LPS induced inflammation. Heatmaps showing expression patterns of gene sets derived from (A) GO:0006954 (inflammatory response), (B) GO:0002526 (acute inflammatory response), and (C) GO:0032496 (response to LPS). (PDF 111 kb)

**Additional file 14: Figure S6.** Cross-validated representative profiles of genes responsive to LPS stimulation. Genes showing differential expression post LPS injection compared to baseline were cross-validated by using independent time-series gene expression microarray data studying pigs’ whole blood responses to LPS [23]. In Terenina et al. [23], DEGs showed delayed responses likely because a lower dosage of LPS was injected and/or injection sites were different. The y-axis shows the *log*_*2*_ fold change of gene expression at each time point relative to baseline, estimated by *DESeq2* or *limma*. Smoothed expression profiles of individual genes per line were inferred by using LOWESS (Locally Weighted Scatterplot Smoothing). HRFI, high-RFI line; LRFI, low-RFI line; Normal, pigs not selected for RFI [23]. (PDF 101 kb)

**Additional file 15: Table S8.** GO terms overrepresented among DEGs post LPS injection compared to baseline. (XLSX 412 kb)

**Additional file 16: Figure S7.** Different MHC class II gene expression patterns between the low-RFI and high-RFI lines. Expression patterns of 17 genes associated with GO:0002504 are shown, with 6 MHC class II genes significantly differentially expressed between the two lines at 24 hpi. (PDF 32 kb)

**Additional file 17: Figure S8.** KEGG pathway-derived gene sets enriched among DEGs post LPS injection relative to baseline. Shown are gene sets significantly enriched under at least one condition (*q* < 0.01). (A-C) Enriched gene sets derived from KEGG signaling pathways (A), metabolic pathways (B), and disease pathways (C), respectively. Values displayed as heatmaps are *log*_*10*_(*q* value) or -*log*_*10*_(*q* value), respectively, if gene sets were enriched among genes of lower or higher expression levels in the low-RFI animals than in the high-RFI animals at a given time point. For better visualization, values greater than 10 or less than − 10 were set to 10 or − 10, respectively. HRFI, high-RFI line; LRFI, low-RFI line. (PDF 44 kb)

## Data Availability

RNA-seq data are available in ArrayExpress under accession number: [E-MTAB-5606, https://www.ebi.ac.uk/arrayexpress/]. Gene expression microarray data were generated by Terenina et al. [23] and are available from the NCBI GEO under accession number: [GSE107487, https://www.ncbi.nlm.nih.gov/geo/]. Metadata for RNA-seq samples are available in Table S1. Rectal temperature data, full CBC data, cytokine data and RT-qPCR data are available upon request.

## Abbreviations

AIC: Akaike Information Criterion
AR: Autoregressive
ARH: Heterogeneous Autoregressive
BH: Benjamini-Hochberg
BW: Body Weight
CBC: Complete Blood Count
CFA: Complete Freund’s Adjuvant
cpm: Count Per Million mapped reads
CS: Compound Symmetry
CSH: Heterogeneous Compound Symmetry
DEG: Differentially Expressed Gene
EDTA: Ethylenediaminetetraacetic Acid
ENCODE: Encyclopedia of DNA Elements
FDR: False Discovery Rate
GO: Gene Ontology
GOA: Gene Ontology Overrepresentation Analysis
GSEA: Gene Set Enrichment Analysis
GTF: Gene Transfer Format
hpi: Hours Post initial Injection
IACUC: Institutional Animal Care and Use Committee
IgG: immunoglobulin gamma
INRA: National Institute for Agricultural Research
ISU: Iowa State University
KEGG: Kyoto Encyclopedia of Genes and Genomes
LD: *longissimus dorsi*
LPS: Lipopolysaccharide
MHC: Major Histocompatibility Complex
NCBI GEO: the Gene Expression Omnibus database at the National Center for Biotechnology Information
NLR: Neutrophils/Lymphocytes Ratio
PBMC: Periphral Blood Mononuclear Cell
PCA: Principal Component Analysis
PRRSV: Porcine Reproductive and Respiratory Syndrome Virus
RBC: Red Blood Cell
RFI: Residual Feed Intake
RIN: RNA Integrity Number
RNA-seq: RNA sequencing
ROS: Reactive Oxygen Species
RT-qPCR: Reverse Transcription-quantitative PCR
SP: Spatial Power
STEM: Short Timer-series Expression Miner
UN: Unstructured
WBC: White Blood Cell
