# Supplementary material for: Acute systemic inflammatory response to lipopolysaccharide stimulation in pigs divergently selected for residual feed intake
Source: BMC Genomics. 2019 Oct 11;20:728. doi: 10.1186/s12864-019-6127-x (PMC6792331; doi:10.1186/s12864-019-6127-x)
Supplement: Supplementary file 3 — Additional file 3: Supplementary methods. This file contains parameter settings for STEM analyses. (DOCX 27 kb) [file 12864_2019_6127_MOESM3_ESM.docx]

Supplementary methods

Reanalysis of microarray data for pig whole blood transcriptomic response to LPS

Raw Microarray expression data and its metadata under accession number GSE107487 for pig whole blood transcriptomic response to LPS stimulation [1] were retrieved from the NCBI GEO database. For details of the experimental design, see Terenina *et al*. [1]. Briefly, 30 female 8 week-old pigs from two cohorts were injected in the neck muscle with 15 μg/kg BW of LPS (*E. coli* serotype O55:B5, Sigma-Aldrich, St. Louis, MO, USA). Blood samples were collected immediately before injection (0 hpi), and at 1, 4, and 24 hpi. Transcriptomic responses were profiled by using the Agilent SurePrint G3 porcine microarrays (GPL16524). The probesets were initially filtered as described by Terenina *et al*. [1]. The signal intensities per probeset were *log*-transformed and quantile normalized. Although Terenina *et al*. [1] suggested adjusting the signal intensities for only batch effects introduced by 30 different hybridization and washing batches, we found that the surrogate variables-based method better accounted for the hidden, nuisance variations. Seventeen surrogate variables accounting for hidden batch effects and other nuisance variations were identified by using the R/Bioconductor package *sva* (v3.28.0) [2] and considered in the linear models for differential expression analysis using a full model with an intercept and a time effect, and a reduced model including only the intercept term. Probesets showing significant differential expression with |*log*_2_(fold change)| > *log*_2_(1.2) and *q* < 0.05 at each time point post LPS injection compared to baseline were identified by using the R/Bioconductor *limma* package [3], with a linear model including normalized, *log*-transformed expression values as dependent variables, and time and the 17 surrogate variable as independent variables. The cohort effect was not considered by Terenina *et al*. [1] in their original analysis and the cohort information is not included in the metadata. Thus, we did not consider the cohort effect in our reanalysis either. In addition, we did not consider the within-animal correlation structure of gene expression in our reanalysis, although it is possible to account for that given that 30 pigs were used in the study. The reason was that we wanted to apply differential expression analysis methods as similar as possible to both the microarray [1] and the RNA-seq (this study) gene expression data to make the results comparable. Multiple testing correction was performed by using the BH method [4].

Normalized, *log*-transformed expression values of multiple probesets uniquely mapping to the same individual genes were collapsed by using the *collapseRows* function from the R package *WGCNA* [5] with the method of “MaxMean”. Expression values after collapsing were compared to adjusted *log(cpm)* expression values measured in response to LPS stimulation in this study.

Parameter settings for STEM-based clustering analysis

#Main Input:

Data_File C:\Users\Haibo\Desktop\Pig LPS acute response reanalysis with Sus scrofa11.1\STEM.6sva.only\Pig 1 DESeq2 norm voom adj6 sva logcpm.txt

Gene_Annotation_Source User provided

Gene_Annotation_File C:\Users\Haibo\Desktop\Pig LPS acute response reanalysis with Sus scrofa11.1\Ssc11.1.GO-BP.mt10.lt500.terms.STEM.format.txt

Cross_Reference_Source User provided

Cross_Reference_File

Gene_Location_Source User provided

Gene_Location_File

Clustering_Method[STEM Clustering Method, K-means] STEM Clustering Method

Maximum_Number_of_Model_Profiles 100

Maximum_Unit_Change_in_Model_Profiles_between_Time_Points 10

Normalize_Data[Log normalize data, Normalize data, No normalization/add 0] Normalize data

Spot_IDs_included_in_the_data_file true

#Repeat data

Repeat_Data_Files(comma delimited list) C:\Users\Haibo\Desktop\Pig LPS acute response reanalysis with Sus scrofa11.1\STEM.6sva.only\Pig 2 DESeq2 norm voom adj6 sva logcpm.txt,C:\Users\Haibo\Desktop\Pig LPS acute response reanalysis with Sus scrofa11.1\STEM.6sva.only\Pig 3 DESeq2 norm voom adj6 sva logcpm.txt,C:\Users\Haibo\Desktop\Pig LPS acute response reanalysis with Sus scrofa11.1\STEM.6sva.only\Pig 4 DESeq2 norm voom adj6 sva logcpm.txt,C:\Users\Haibo\Desktop\Pig LPS acute response reanalysis with Sus scrofa11.1\STEM.6sva.only\Pig 5 DESeq2 norm voom adj6 sva logcpm.txt,C:\Users\Haibo\Desktop\Pig LPS acute response reanalysis with Sus scrofa11.1\STEM.6sva.only\Pig 6 DESeq2 norm voom adj6 sva logcpm.txt,C:\Users\Haibo\Desktop\Pig LPS acute response reanalysis with Sus scrofa11.1\STEM.6sva.only\Pig 7 DESeq2 norm voom adj6 sva logcpm.txt,C:\Users\Haibo\Desktop\Pig LPS acute response reanalysis with Sus scrofa11.1\STEM.6sva.only\Pig 8 DESeq2 norm voom adj6 sva logcpm.txt

Repeat_Data_is_from[Different time periods,The same time period] The same time period

#Comparison Data:

Comparison_Data_File

Comparison_Repeat_Data_Files(comma delimited list)

Comparison_Repeat_Data_is_from[Different time periods,The same time period] Different time periods

Comparison_Minimum_Number_of_genes_in_intersection 5

Comparison_Maximum_Uncorrected_Intersection_pvalue 0.005

#Filtering:

Maximum_Number_of_Missing_Values 3

Minimum_Correlation_between_Repeats 0.5

Minimum_Absolute_Log_Ratio_Expression 0.59

Change_should_be_based_on[Maximum-Minimum,Difference From 0] Difference From 0

Pre-filtered_Gene_File

#Model Profiles

Maximum_Correlation 1.0

Number_of_Permutations_per_Gene 50

Maximum_Number_of_Candidate_Model_Profiles 1000000

Significance_Level 0.05

Correction_Method[Bonferroni,False Discovery Rate,None] False Discovery Rate

Permutation_Test_Should_Permute_Time_Point_0 false

#Clustering Profiles:

Clustering_Minimum_Correlation 0.6

Clustering_Minimum_Correlation_Percentile 0.0

#Gene Annotations:

Category_ID_File

Include_Biological_Process true

Include_Molecular_Function false

Include_Cellular_Process false

Only_include_annotations_with_these_evidence_codes

Only_include_annotations_with_these_taxon_IDs

#GO Analysis:

Multiple_hypothesis_correction_method_enrichment[Bonferroni,Randomization] Randomization

Minimum_GO_level 5

GO_Minimum_number_of_genes 10

Number_of_samples_for_randomized_multiple_hypothesis_correction 500

#Interface Options

Gene_display_policy_on_main_interface[Do not display,Display only selected,Display all] Display all

Gene_Color(R,G,B) 204,51,0

Display_Model_Profile true

Display_Profile_ID true

Display_details_when_ordering true

Show_Main_Y-axis_gene_tick_marks false

Main_Y-axis_gene_tick_interval 1.0

Y-axis_scale_for_genes_on_main_interface_should_be[Gene specific,Profile specific,Global] Gene specific

Scale_should_be_based_on_only_selected_genes true

Y-axis_scale_on_details_windows_should_be[Determined automatically,Fixed] Determined automatically

Y_Scale_Min -3.0

Y_Scale_Max 3.0

Tick_interval 1.0

X-axis_scale_should_be[Uniform, Based on real time] Based on real time

References

1. Terenina E, Sautron V, Ydier C, Bazovkina D, Sevin-Pujol A, Gress L, Lippi Y, Naylies C, Billon Y, Liaubet L *et al*: **Time course study of the response to LPS targeting the pig immune gene networks**. *BMC Genomics* 2017, **18**(1):988.

2. Leek JT, Johnson WE, Parker HS, Jaffe AE, Storey JD: **The sva package for removing batch effects and other unwanted variation in high-throughput experiments**. *Bioinformatics* 2012, **28**(6):882-883.

3. Ritchie ME, Phipson B, Wu D, Hu Y, Law CW, Shi W, Smyth GK: **limma powers differential expression analyses for RNA-sequencing and microarray studies**. *Nucleic Acids Res* 2015, **43**(7):e47.

4. Benjamini Y, Hochberg Y: **Controlling the false discovery rate: A practical and powerful approach to multiple testing**. *J R Stat Soc Series B Stat Methodol* 1995, **57**(1):289-300.

5. Langfelder P, Horvath S: **WGCNA: an R package for weighted correlation network analysis**. *BMC Bioinformatics* 2008, **9**:559.
