# Supplementary figures and images for: Acute systemic inflammatory response to lipopolysaccharide stimulation in pigs divergently selected for residual feed intake
Source: BMC Genomics. 2019 Oct 11;20:728. doi: 10.1186/s12864-019-6127-x (PMC6792331; doi:10.1186/s12864-019-6127-x)

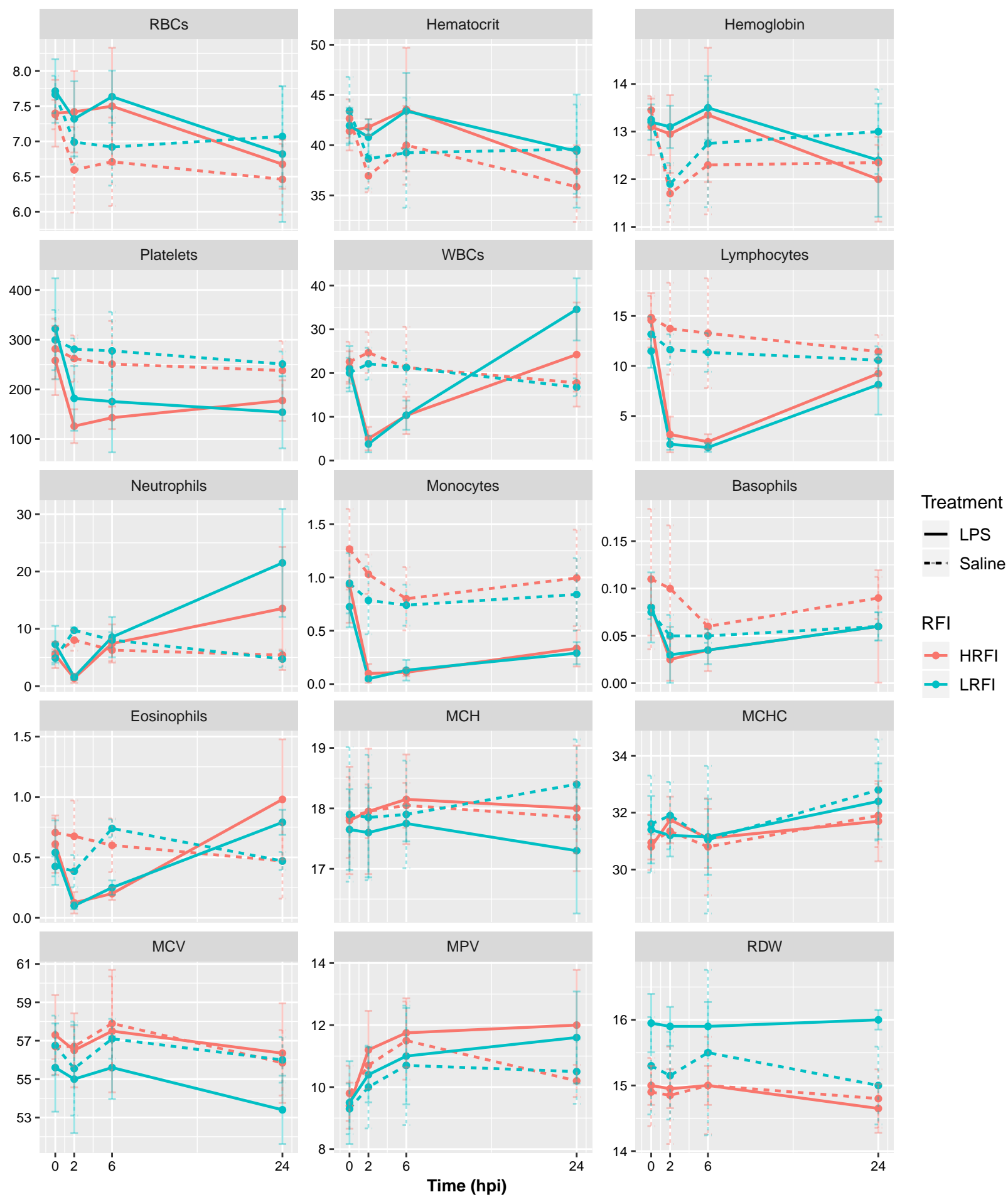

Supplement: Supplementary file 5 — Additional file 5: Figure S1. Profiles of CBC parameters during the 24-h time course. Shown are medians of measurement of each CBC parameter ± median absolute deviation (MAD). The units for the y-axes are 106/μl (RBCs), % (Hematocrit), g/dl (Hemoglobin), 103/μl (Platelet), 103/μl (WBCs), 103/μl (Lymphocytes, Neutrophils, Monocytes, Basophils and Eosinophils), pg (MCH), g/dl (MCHC), fl (MCV, MPV) and % (RDW). HRFI, high-RFI line; LRFI, low-RFI line. (PDF 17 kb) [file 12864_2019_6127_MOESM5_ESM.pdf]

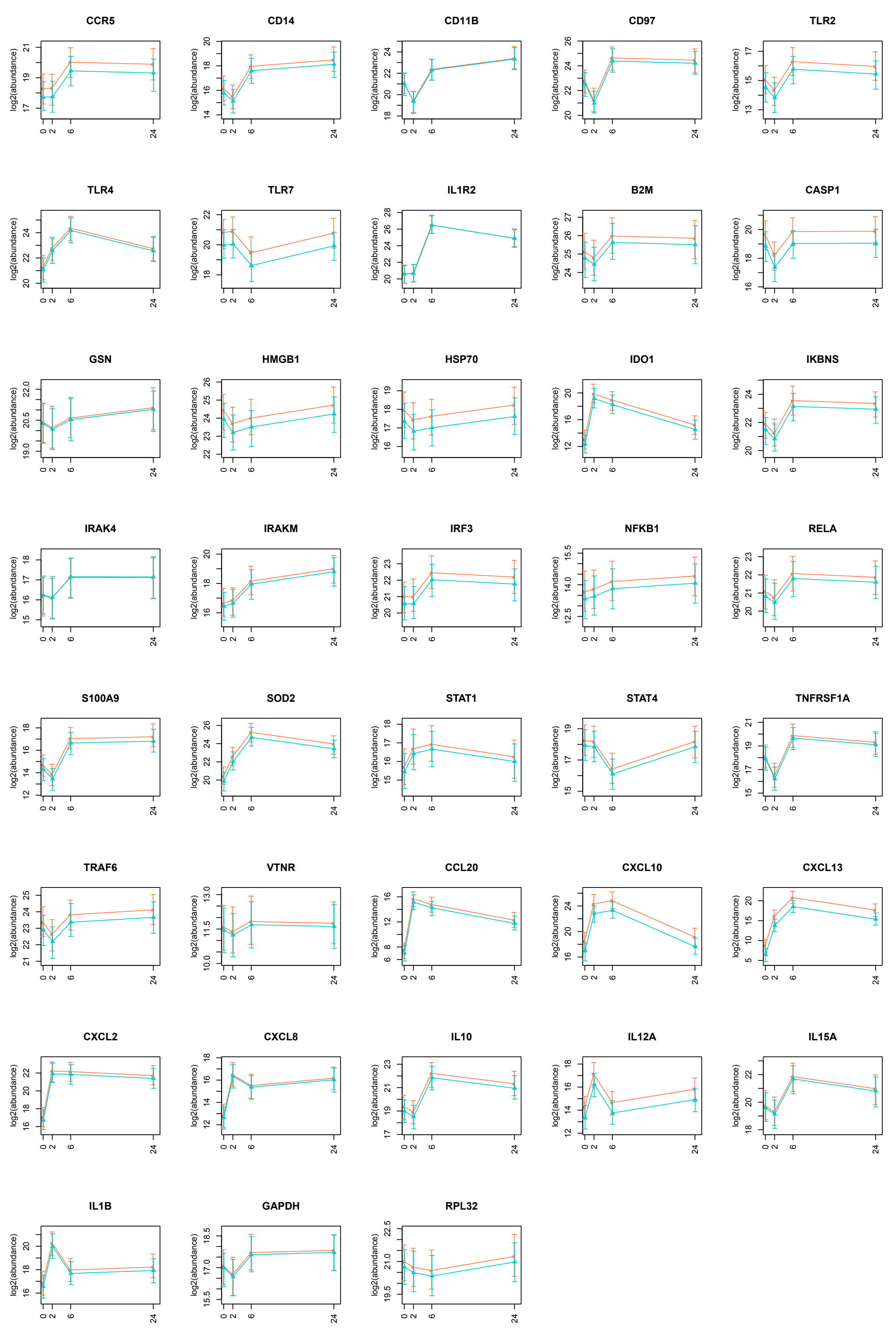

Supplement: Supplementary file 8 — Additional file 8: Figure S2. Expression profiles of inflammation-related genes determined by RT-qPCR. Shown are least square means of log2(abundance) ± 95% confidence intervals of 36 genes, and two assumed internal reference genes (RPL32 and GAPDH). Notably, the expression levels of the assumed internal references were not stable over the time course of the study. HRFI, high-RFI line; LRFI, low-RFI line. (PDF 144 kb) [file 12864_2019_6127_MOESM8_ESM.pdf]

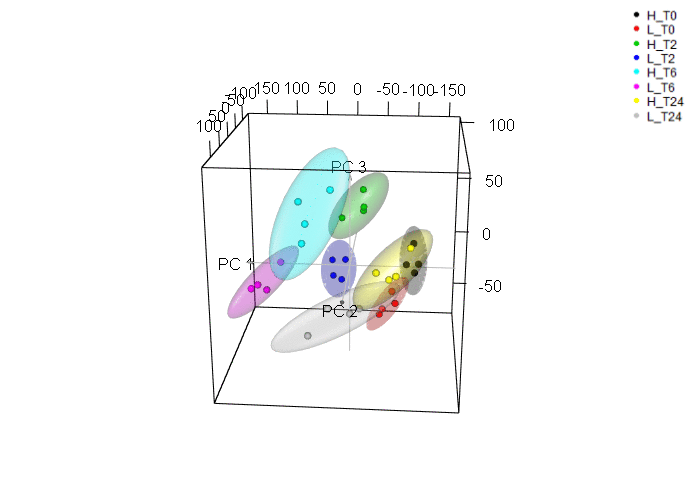

Supplement: Supplementary file 9 — Additional file 9: Figure S3. 3D-PCA plots showing the relationship of RNA-seq samples. H, high-RFI line; L, low-RFI line; Tx, x hpi. H_T0, samples at baseline from the high-RFI line treated with LPS. (GIF 2332 kb) [file 12864_2019_6127_MOESM9_ESM.gif]

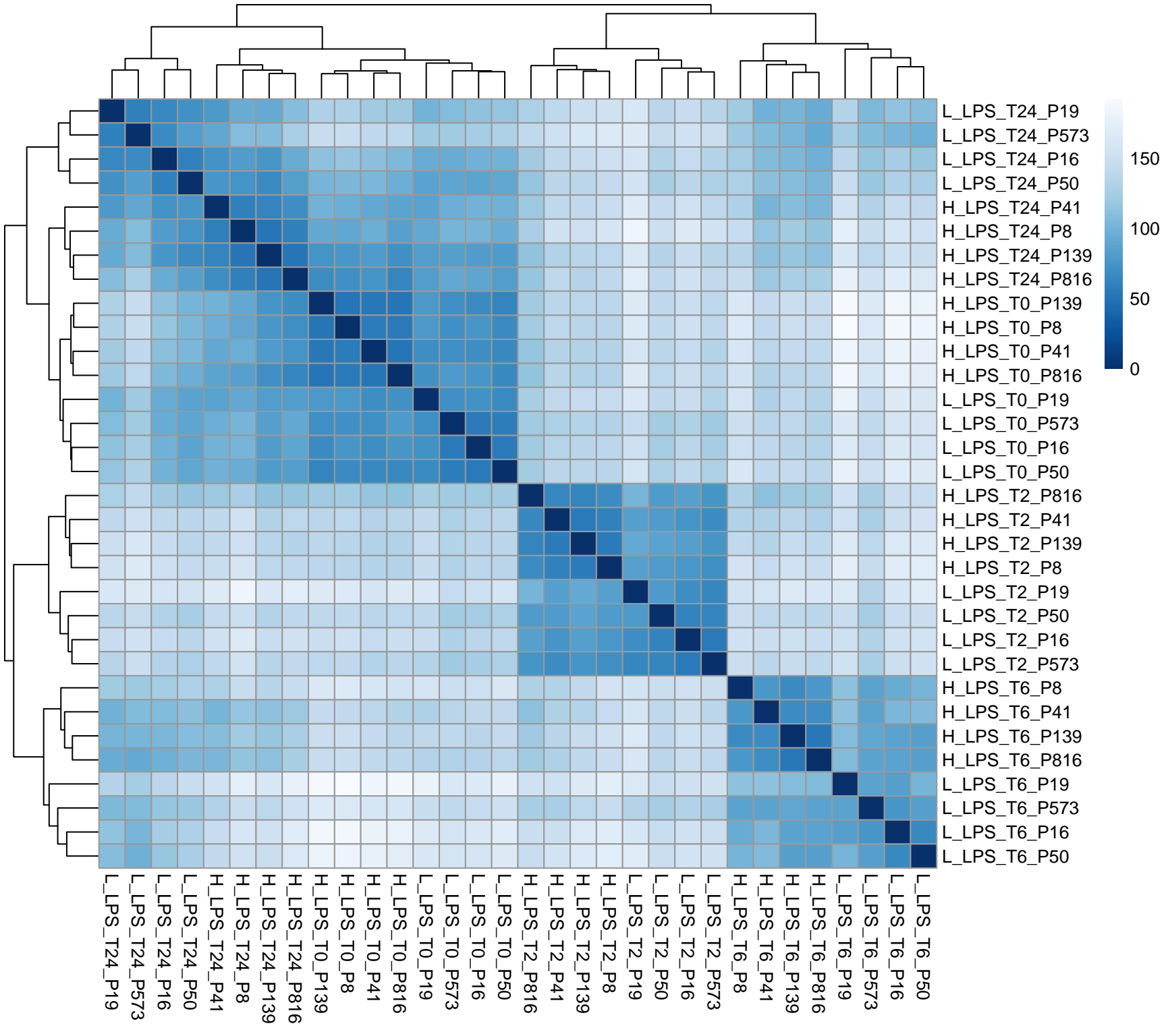

Supplement: Supplementary file 10 — Additional file 10: Figure S4. Heatmap showing sample similarities. Euclidian distances between samples were calculated based on adjusted log2(cpm) gene expression. Samples were then hierarchically clustered using the complete linkage clustering method. Pheatmap was used to generate the heatmap. H, high-RFI line; L, low-RFI line; Tx, x hpi. H_LPS_T2_P41, 2 hpi sample of a high-RFI pig with ear tag 41treated with LPS. (PDF 14 kb) [file 12864_2019_6127_MOESM10_ESM.pdf]

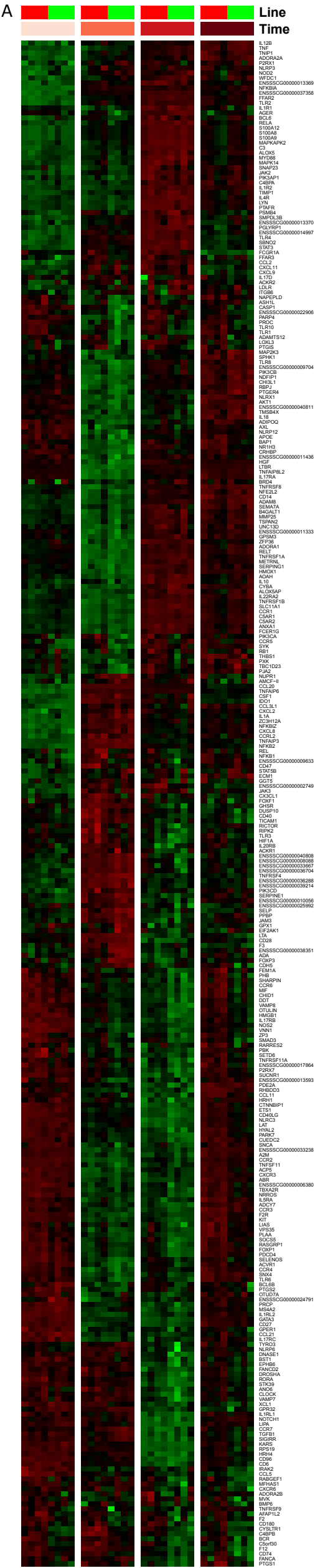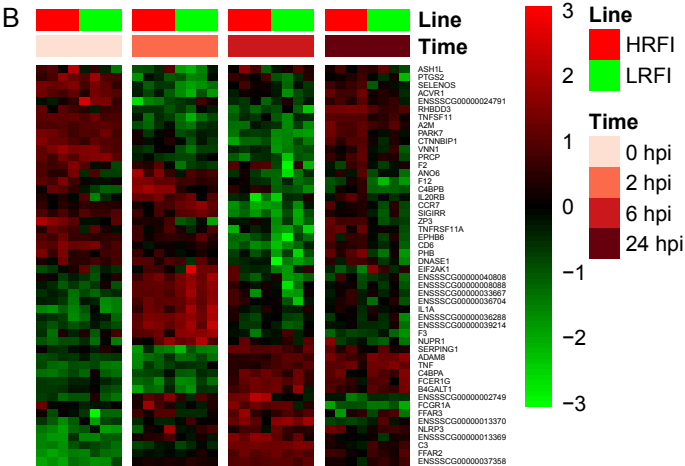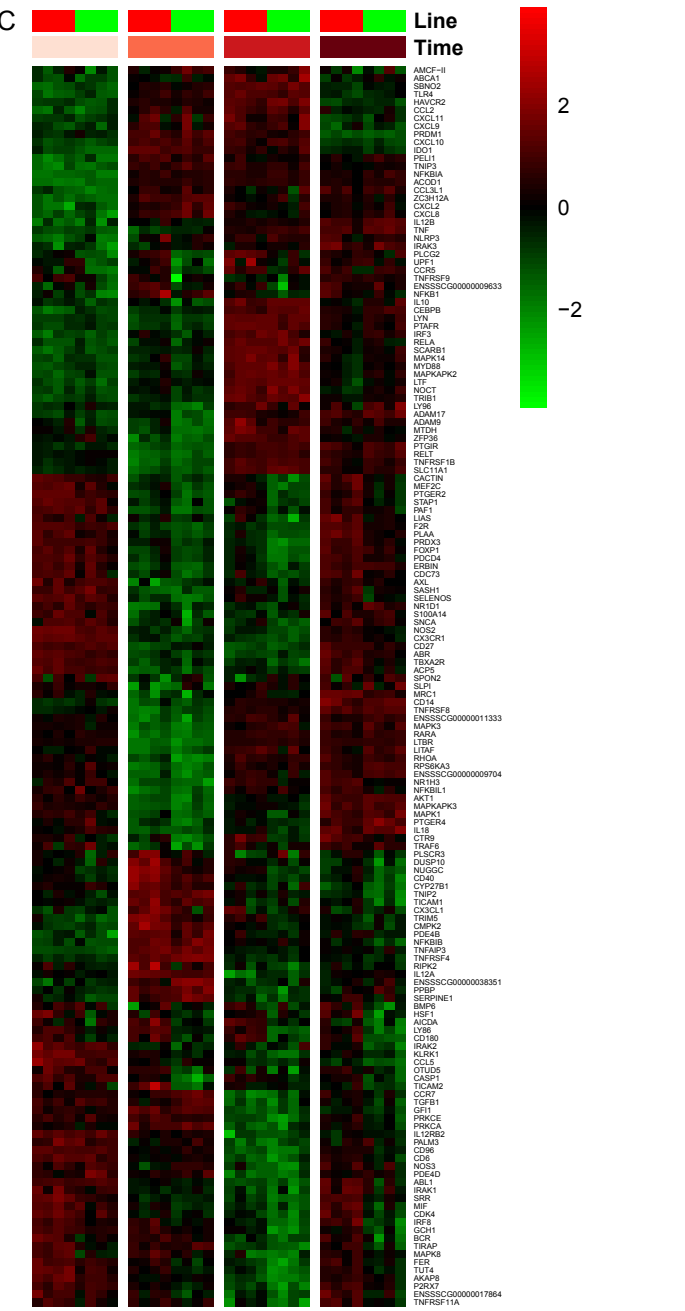

Supplement: Supplementary file 13 — Additional file 13: Figure S5. Expression patterns of gene sets derived from GO-BP terms directly related to LPS induced inflammation. Heatmaps showing expression patterns of gene sets derived from (A) GO:0006954 (inflammatory response), (B) GO:0002526 (acute inflammatory response), and (C) GO:0032496 (response to LPS). (PDF 111 kb) [file 12864_2019_6127_MOESM13_ESM.pdf]

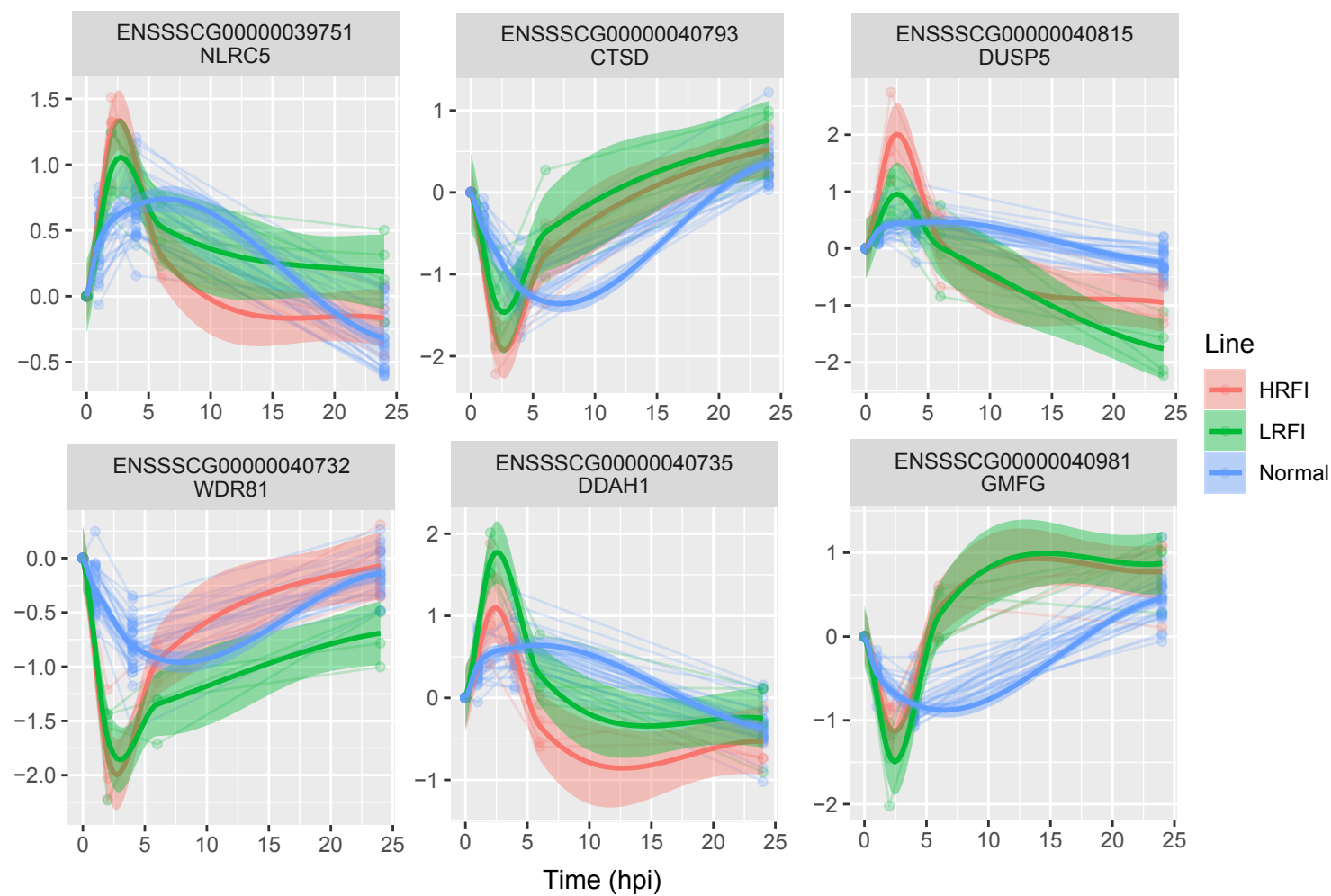

Supplement: Supplementary file 14 — Additional file 14: Figure S6. Cross-validated representative profiles of genes responsive to LPS stimulation. Genes showing differential expression post LPS injection compared to baseline were cross-validated by using independent time-series gene expression microarray data studying pigs’ whole blood responses to LPS [23]. In Terenina et al. [23], DEGs showed delayed responses likely because a lower dosage of LPS was injected and/or injection sites were different. The y-axis shows the log2 fold change of gene expression at each time point relative to baseline, estimated by DESeq2 or limma. Smoothed expression profiles of individual genes per line were inferred by using LOWESS (Locally Weighted Scatterplot Smoothing). HRFI, high-RFI line; LRFI, low-RFI line; Normal, pigs not selected for RFI [23]. (PDF 101 kb) [file 12864_2019_6127_MOESM14_ESM.pdf]

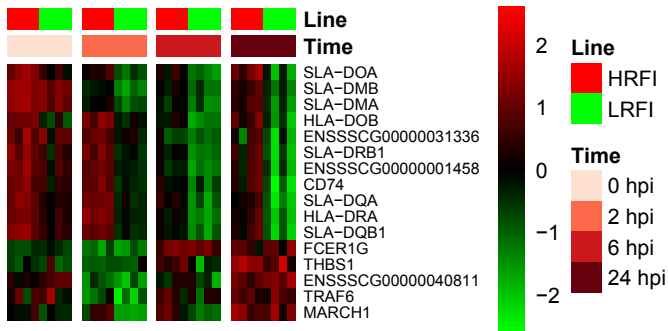

Supplement: Supplementary file 16 — Additional file 16: Figure S7. Different MHC class II gene expression patterns between the low-RFI and high-RFI lines. Expression patterns of 17 genes associated with GO:0002504 are shown, with 6 MHC class II genes significantly differentially expressed between the two lines at 24 hpi. (PDF 32 kb) [file 12864_2019_6127_MOESM16_ESM.pdf]

A

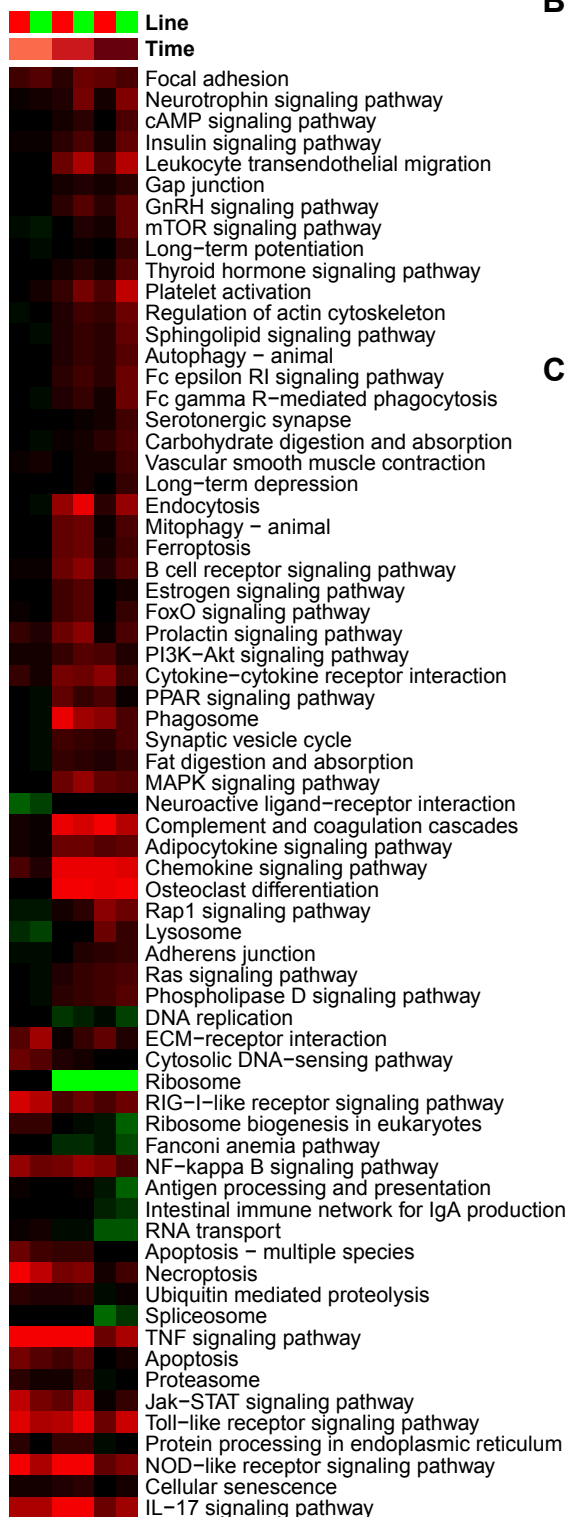

B

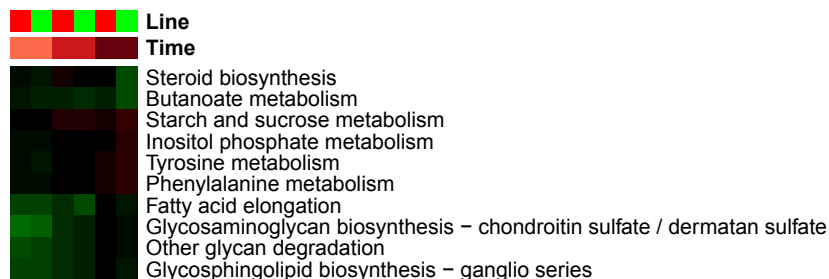

C

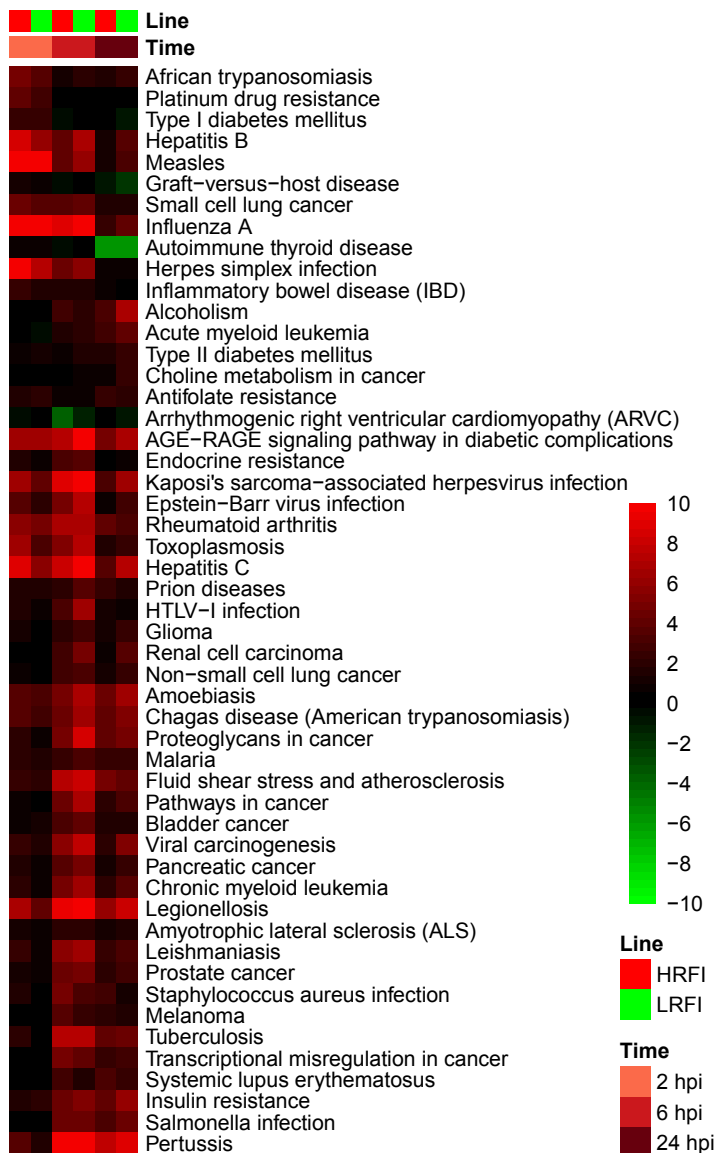

Supplement: Supplementary file 17 — Additional file 17: Figure S8. KEGG pathway-derived gene sets enriched among DEGs post LPS injection relative to baseline. Shown are gene sets significantly enriched under at least one condition (q < 0.01). (A-C) Enriched gene sets derived from KEGG signaling pathways (A), metabolic pathways (B), and disease pathways (C), respectively. Values displayed as heatmaps are log10(q value) or -log10(q value), respectively, if gene sets were enriched among genes of lower or higher expression levels in the low-RFI animals than in the high-RFI animals at a given time point. For better visualization, values greater than 10 or less than − 10 were set to 10 or − 10, respectively. HRFI, high-RFI line; LRFI, low-RFI line. (PDF 44 kb) [file 12864_2019_6127_MOESM17_ESM.pdf]
